# Modelling and DNA topology of compact 2-start and 1-start chromatin fibres

**DOI:** 10.1093/nar/gkz495

**Published:** 2019-06-20

**Authors:** Chenyi Wu, Andrew Travers

**Affiliations:** 1 Molecular Biophysics Laboratories, School of Biological Sciences, University of Portsmouth, Portsmouth PO1 2DY, UK; 2 MRC Laboratory of Molecular Biology, Francis Crick Avenue, Cambridge Biomedical Campus, Cambridge CB2 0QH, UK; 3 Department of Biochemistry, University of Cambridge, Tennis Court Road, Cambridge CB2 1GA, UK

## Abstract

We have investigated the structure of the most compact 30-nm chromatin fibres by modelling those with 2-start or 1-start crossed-linker organisations. Using an iterative procedure we obtained possible structural solutions for fibres of the highest possible compaction permitted by physical constraints, including the helical repeat of linker DNA. We find that this procedure predicts a quantized nucleosome repeat length (NRL) and that only fibres with longer NRLs (≥197 bp) can more likely adopt the 1-start organisation. The transition from 2-start to 1-start fibres is consistent with reported differing binding modes of the linker histone. We also calculate that in 1-start fibres the DNA constrains more torsion (as writhe) than 2-start fibres with the same NRL and that the maximum constraint obtained is in accord with previous experimental results. We posit that the coiling of the fibre is driven by overtwisting of linker DNA which, in the most compact forms - for example, in echinoderm sperm and avian erythrocytes - could adopt a helical repeat of ∼10 bp/turn. We argue that *in vivo* the total twist of linker DNA could be modulated by interaction with other abundant chromatin-associated proteins and by epigenetic modifications of the C-terminal tail of linker histones.

## INTRODUCTION

Elucidating the mechanics of chromatin condensation remains a fundamental challenge in molecular biology. Original EM studies on isolated chromatin revealed ‘beads on a string’ structures indicating nucleosomes were connected by the linker DNA. Filaments of varying diameter were also observed in partially folded chromatin, giving rise to the notion of the 30-nm fibre. These findings suggested that chromatin organisation *in vivo* results from a complex interplay of hierarchical folding pathways (1,2).

In the 30-nm fibre an array of nucleosomes is coiled into one or more helical stacks with the linker histone located in the interior of the fibre. Discussions of its structure have centred mainly on two characteristics - whether there are one or two stacks (respectively 1-start and 2-start helices) and whether the trajectory of the linker DNA between consecutive nucleosomes crosses the interior of the fibre (the crossed-linker model) or approximately continues the tightly bent path of nucleosomal DNA (the solenoid model). Whereas the crossed-linker model is compatible with both 1-start and 2-start structures (3,4) the solenoid model predicts a 1-start structure (5).

It is sometimes forgotten that in their original seminal description of the 30-nm fibre Finch and Klug (5) observed two classes of chromatin fibre, one with a low and the other with a higher pitch angle. These, they argued, were consistent with respectively 1-start and 2-start organisations. Subsequent X-ray diffraction and circular dichroism studies (6–9) confirmed a low pitch angle associated with 30-nm fibre preparations. However, more recently several crystallographic and cryo-EM studies on reconstituted fibres have strongly supported a higher pitch 2-start structure (10–13). These structures have packing densities of 3.9–7 nucleosomes/11 nm. In contrast, values of 10–15 nucleosomes/11 nm are attained by certain isolated chromatin fibres mostly under conditions of higher ionic strength (14–18). Similarly, Robinson *et al.* (19) and Routh *et al.* (20) reported values of 9–15 nucleosomes/11nm for reconstituted fibres formed *in vitro* on long (187–237 bp) but not short (167 bp) quantized nucleosome repeat lengths (NRLs).

To accommodate all these apparently conflicting observations we have here combined a detailed structural and topological model with a single unifying compaction mechanism. In particular, we recently proposed that fibres of high packing density are formed by the interdigitation of the two nucleosome stacks in a loosely packed 2-start crossed-linker fibre to assume a 1-start configuration, resembling a low pitch solenoid but retaining the more energetically favoured trajectory for the linker DNA (4). In this work to validate and refine our model we have constructed a series of 2- and 1-start fibres by exploring the utility of model building. The DNA packing density (per unit fibre volume) was expressed as a function of geometric variables, which were iterated for convergence on maximum packing. Physical constraints were formulated according to published data including the structure of a crystallized tetranucleosome (10) to facilitate the observed nucleosome stacking. From the DNA trajectory, we computed the writhe of fibre DNA for different NRLs (21–23).

We argue that with increasing NRL the two nucleosome stacks in a 2-start fibre could merge to form the 1-start configuration (see movie S1 in Supplementary Data) for greater stability. Here, a concomitant slight increase in fibre diameter and a reduction in the helical pitch of the fibre means that a similar curvature of the nucleosome stack is maintained. Moreover, the need for a correct register between adjacent octamers, including a relative rotation around their common axis, implies quantised linkers and different binding modes of linker histone respectively in 2- and 1-start structures. We find in most compact structures that the 1-start fibres constrain more negative writhe than 2-start. Importantly, the calculated topological change agrees closely with the experimentally determined external torsion required for maximal compaction (24). We further argue that the intercalation resulting in the formation of the 1-start configuration facilitates a closer approach of adjacent linker DNAs possibly enabling a phase transition in DNA packing.

It is well established that the coiling of 30-nm fibres is strongly dependent on ionic strength (17,25,26). Previous modelling studies have largely emphasized the role of the electrolyte in reducing repulsion between DNA duplexes in close proximity. However cations, especially Mg^2+^ and Co^3+^ widely used for fibre compaction (8), also overtwist DNA (27–29). We have therefore asked whether this overtwisting is relevant to folding process. We find that electrolyte-dependent overtwisting of DNA correlates with observed fibre densities. In addition the C-terminal tail of linker histones contains multiple positive charges and can by itself induce DNA condensation to form a chiral cholesteric structure (30). To account for any additional overtwisting dependent on the C-terminal tail we have estimated that in the most compact fibres with the longest NRLs - for example, those in echinoderm sperm and avian erythrocytes - the helical repeat of linker DNA to be ∼10 bp/turn (Supplementary data S2). Our model implies that the overtwisting of the linker DNA and hence the chirality of chromatin fibres can potentially be fine-tuned *in vivo* by epigenetic modification of the tail - for example, the CDK-mediated phosphorylation of SPKK sequences. In addition, especially in interphase nuclei, interaction with other abundant chromatin-associated proteins - for example, HMGB proteins - could potentially untwist the linker DNA (31).

## MATERIALS AND METHODS

### Finding the path of nucleosomal DNA

The structure of a chromatosome (PDB ID: 4LQC) was realigned in Pymol by means of least square fit using a transformation matrix (${\rm{A}}$):

$$\begin{equation*}{\rm{A}} \cdot \left[ {\begin{array}{@{}*{4}{c}@{}} {{{\rm{x}}_{20}}}&{{{\rm{x}}_{21}}}& \ldots &{{{\rm{x}}_{148}}}\\ {{{\rm{y}}_{20}}}&{{{\rm{y}}_{21}}}& \ldots &{{{\rm{y}}_{148}}}\\ {{{\rm{z}}_{20}}}&{{{\rm{z}}_{21}}}& \ldots &{{{\rm{z}}_{148}}}\\ 1&1& \ldots &1 \end{array}} \right] \approx \left[ {\begin{array}{@{}*{4}{c}@{}} {{{{\rm{x^{\prime}}}}_{20}}}&{{{{\rm{x^{\prime}}}}_{21}}}& \ldots &{{{{\rm{x^{\prime}}}}_{148}}}\\ {{{{\rm{y^{\prime}}}}_{20}}}&{{{{\rm{y^{\prime}}}}_{21}}}& \ldots &{{{{\rm{y^{\prime}}}}_{148}}}\\ {{{{\rm{z^{\prime}}}}_{20}}}&{{{{\rm{z^{\prime}}}}_{21}}}& \ldots &{{{{\rm{z^{\prime}}}}_{148}}}\\ 1&1& \ldots &1 \end{array}} \right]\end{equation*}$$

where

$$\begin{equation*} \begin{array}{@{}*{4}{c}@{}} {{\rm{A\ }} = \left[ {\begin{array}{@{}*{4}{c}@{}} {{\rm{cos}}\left( {{{\rm{\theta }}_1}} \right)}&{ - {\rm{sin}}\left( {{{\rm{\theta }}_1}} \right)}&0&0\\ {{\rm{sin}}\left( {{{\rm{\theta }}_1}} \right)}&{{\rm{cos}}\left( {{{\rm{\theta }}_1}} \right)}&0&0\\ 0&0&1&0\\ 0&0&0&1 \end{array}} \right]{\rm{\ }} \cdot \left[ {\begin{array}{@{}*{4}{c}@{}} {{\rm{cos}}\left( {{{\rm{\theta }}_2}} \right)}&0&{ - {\rm{sin}}\left( {{{\rm{\theta }}_2}} \right)}&0\\ 0&1&0&0\\ {{\rm{sin}}\left( {{{\rm{\theta }}_2}} \right)}&0&{{\rm{cos}}\left( {{{\rm{\theta }}_2}} \right)}&0\\ 0&0&0&1 \end{array}} \right] \cdot } {\left[ {\begin{array}{@{}*{4}{c}@{}} 1&0&0&0\\ 0&{{\rm{cos}}\left( {{{\rm{\theta }}_3}} \right)}&{ - {\rm{sin}}\left( {{{\rm{\theta }}_3}} \right)}&0\\ 0&{{\rm{sin}}\left( {{{\rm{\theta }}_3}} \right)}&{{\rm{cos}}\left( {{{\rm{\theta }}_3}} \right)}&0\\ 0&0&0&1 \end{array}} \right] \cdot \left[ {\begin{array}{@{}*{4}{c}@{}} 1&0&0&{{{\rm{x}}_{\rm{v}}}}\\ 0&1&0&{{{\rm{y}}_{\rm{v}}}}\\ 0&0&1&{{{\rm{z}}_{\rm{v}}}}\\ 0&0&0&1 \end{array}} \right]} \end{array} \end{equation*}$$

**Figure 1.**
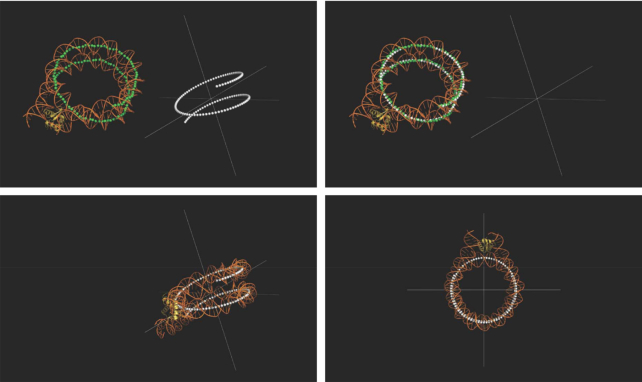
Defining the path of nucleosomal DNA. The path of the central 129 bp of the nucleosomal DNA (PDB ID: 4LQC) was shown in green, while a theoretical path was generated at the Cartesian origin shown in white. The nucleosome was realigned at the origin by means of transformation such that the y and z axes (z axis being normal to the plane of view) correspond to the nucleosome dyad and superhelical axes respectively. Here the dyad axis is also the 2-fold symmetry axis of the nucleosomal DNA.

$( {{\rm{x}},{\rm{\ y}},{\rm{\ z}}} )$ are position coordinates of individual base pairs acquired by calculating midpoints between opposing phosphorus atoms over the central 129 bp of the nucleosomal DNA. Whereas $( {{\rm{x^{\prime}}},{\rm{\ y^{\prime}}},{\rm{\ z^{\prime}}}} )$ are equivalent best fit values on a smooth path around the Cartesian origin (Figure 1) given by:

$$\begin{equation*}\left[ {\begin{array}{@{}*{1}{c}@{}} {\ {\rm{x^{\prime}}} = {\rm{\ }} {\rm{a}} \cdot {\rm{sin}}\left( {{\rm{\omega }} \cdot {\rm{n}}} \right)}\\ {\ {\rm{y^{\prime}}} = {\rm{\ a}} \cdot {\rm{cos}}\left( {{\rm{\omega }} \cdot {\rm{n}}} \right)}\\ {\ {\rm{z^{\prime}}} = {\rm{\ q}} \cdot {\rm{\omega }} \cdot {\rm{n}}} \end{array}} \right],\,\left( {{\rm{n\ }} = {\rm{\ }}0,{\rm{\ }} \pm 1,{\rm{\ }} \pm 2, \ldots \pm 64} \right)\end{equation*}$$

Solutions for $( {{{\rm{\theta }}_1},{\rm{\ }}{{\rm{\theta }}_2},{\rm{\ }}{{\rm{\theta }}_3},{\rm{\ }}{{\rm{x}}_{\rm{v}}},{\rm{\ }}{{\rm{y}}_{\rm{v}}},{\rm{\ }}{{\rm{z}}_{\rm{v}}},{\rm{\ a}},{\rm{\ \omega }},{\rm{\ q}}} )$ were obtained using Excel Solver formulating:

$$\begin{equation*}{\rm{A}} \cdot \left[ {\begin{array}{@{}*{4}{c}@{}} {{{\rm{x}}_{20}}}&{{{\rm{x}}_{21}}}& \ldots &{{{\rm{x}}_{148}}}\\ {{{\rm{y}}_{20}}}&{{{\rm{y}}_{21}}}& \ldots &{{{\rm{y}}_{148}}}\\ {{{\rm{z}}_{20}}}&{{{\rm{z}}_{21}}}& \ldots &{{{\rm{z}}_{148}}}\\ 1&1& \ldots &1 \end{array}} \right] - \left[ {\begin{array}{@{}*{4}{c}@{}} {{{{\rm{x^{\prime}}}}_{20}}}&{{{{\rm{x^{\prime}}}}_{21}}}& \ldots &{{{{\rm{x^{\prime}}}}_{148}}}\\ {{{{\rm{y^{\prime}}}}_{20}}}&{{{{\rm{y^{\prime}}}}_{21}}}& \ldots &{{{{\rm{y^{\prime}}}}_{148}}}\\ {{{{\rm{z^{\prime}}}}_{20}}}&{{{{\rm{z^{\prime}}}}_{21}}}& \ldots &{{{{\rm{z^{\prime}}}}_{148}}}\\ 1&1& \ldots &1 \end{array}} \right]\end{equation*}$$

where in the resultant matrix from above the summation of squared entries was minimised through iterating solutions for $( {{{\rm{\theta }}_1},{\rm{\ }}{{\rm{\theta }}_2},{\rm{\ }}{{\rm{\theta }}_3},{\rm{\ }}{{\rm{x}}_{\rm{v}}},{\rm{\ }}{{\rm{y}}_{\rm{v}}},{\rm{\ }}{{\rm{z}}_{\rm{v}}},{\rm{\ a}},{\rm{\ \omega }},{\rm{\ q}}} )$.

Additional to the central 129 bp are the entering and leaving DNAs, where each over the 19 bp segment approximates a straight trajectory (Figure 3). Their best fit values (coordinates) were obtained similarly by:

$$\begin{equation*}{\rm{A}} \cdot \left[ {\begin{array}{@{}*{4}{c}@{}} {{{\rm{x}}_2}}&{{{\rm{x}}_3}}& \ldots &{{{\rm{x}}_{19}}}\\ {{{\rm{y}}_2}}&{{{\rm{y}}_3}}& \ldots &{{{\rm{y}}_{19}}}\\ {{{\rm{z}}_2}}&{{{\rm{z}}_3}}& \ldots &{{{\rm{z}}_{19}}}\\ 1&1& \ldots &1 \end{array}} \right] \approx \left[ {\begin{array}{@{}*{4}{c}@{}} {{{{\rm{x^{\prime}}}}_2}}&{{{{\rm{x^{\prime}}}}_3}}& \ldots &{{{{\rm{x^{\prime}}}}_{19}}}\\ {{{{\rm{y^{\prime}}}}_2}}&{{{{\rm{y^{\prime}}}}_3}}& \ldots &{{{{\rm{y^{\prime}}}}_{19}}}\\ {{{{\rm{z^{\prime}}}}_2}}&{{{{\rm{z^{\prime}}}}_3}}& \ldots &{{{{\rm{z^{\prime}}}}_{19}}}\\ 1&1& \ldots &1 \end{array}} \right]\end{equation*}$$

(${{\rm{x}}_1},{{\rm{y}}_1},{\rm{\ }}{{\rm{z}}_1}$ unavailable due to missing 3′ phosphate)

$$\begin{equation*}{\rm{A}} \cdot \left[ {\begin{array}{@{}*{4}{c}@{}} {{{\rm{x}}_{149}}}&{{{\rm{x}}_{150}}}& \ldots &{{{\rm{x}}_{166}}}\\ {{{\rm{y}}_{149}}}&{{{\rm{y}}_{150}}}& \ldots &{{{\rm{y}}_{166}}}\\ {{{\rm{z}}_{149}}}&{{{\rm{z}}_{150}}}& \ldots &{{{\rm{z}}_{166}}}\\ 1&1& \ldots &1 \end{array}} \right] \approx \left[ {\begin{array}{@{}*{4}{c}@{}} {{{{\rm{x^{\prime}}}}_{149}}}&{{{{\rm{x^{\prime}}}}_{150}}}& \ldots &{{{{\rm{x^{\prime}}}}_{166}}}\\ {{{{\rm{y^{\prime}}}}_{149}}}&{{{{\rm{y^{\prime}}}}_{150}}}& \ldots &{{{{\rm{y^{\prime}}}}_{166}}}\\ {{{{\rm{z^{\prime}}}}_{149}}}&{{{{\rm{z^{\prime}}}}_{150}}}& \ldots &{{{{\rm{z^{\prime}}}}_{166}}}\\ 1&1& \ldots &1 \end{array}} \right]\end{equation*}$$

(${{\rm{x}}_{167}},{\rm{\ }}{{\rm{y}}_{167}},{\rm{\ }}{{\rm{z}}_{167}}$ unavailable due to missing 3′ phosphate) where

$$\begin{equation*}\begin{array}{@{}*{1}{c}@{}} {\left[ {\begin{array}{@{}*{1}{c}@{}} {\ {\rm{x^{\prime}}} = {\rm{x}}_0^{{\rm{en}}}{\rm{\ }} + {\rm{n}} \cdot {\rm{v}}_{\rm{x}}^{{\rm{en}}}}\\ {\ {\rm{y^{\prime}}} = {\rm{\ y}}_0^{{\rm{en}}} + {\rm{n}} \cdot {\rm{v}}_{\rm{y}}^{{\rm{en}}}}\\ {\ {\rm{z^{\prime}}} = {\rm{z}}_0^{{\rm{en}}}{\rm{\ }} + {\rm{n}} \cdot {\rm{v}}_{\rm{z}}^{{\rm{en}}}} \end{array}} \right],\,\left( {{\rm{n\ }} = 0,{\rm{\ }}1,{\rm{\ }}2, \ldots 17} \right)}\, {{\rm{for}}\,\left( {{{{\rm{x^{\prime}}}}_2},{\rm{\ }}{{{\rm{y^{\prime}}}}_2},{\rm{\ }}{{{\rm{z^{\prime}}}}_2}} \right) \to \left( {{{{\rm{x^{\prime}}}}_{19}},{\rm{\ }}{{{\rm{y^{\prime}}}}_{19}},{\rm{\ }}{{{\rm{z^{\prime}}}}_{19}}} \right)} \end{array}\end{equation*}$$

$$\begin{equation*}\begin{array}{@{}*{1}{c}@{}} {\left[ {\begin{array}{@{}*{1}{c}@{}} {\ {\rm{x^{\prime}}} = {\rm{x}}_0^{{\rm{ex}}}{\rm{\ }} + {\rm{n}} \cdot {\rm{v}}_{\rm{x}}^{{\rm{ex}}}}\\ {\ {\rm{y^{\prime}}} = {\rm{\ y}}_0^{{\rm{ex}}} + {\rm{n}} \cdot {\rm{v}}_{\rm{y}}^{{\rm{ex}}}}\\ {\ {\rm{z^{\prime}}} = {\rm{z}}_0^{{\rm{ex}}}{\rm{\ }} + {\rm{n}} \cdot {\rm{v}}_{\rm{z}}^{{\rm{ex}}}} \end{array}} \right],\,\left( {{\rm{n}} = 0,{\rm{\ }}1,{\rm{\ }}2, \ldots 17} \right)}\, {{\rm{for}}\,\left( {{{{\rm{x^{\prime}}}}_{149}},{\rm{\ }}{{{\rm{y^{\prime}}}}_{149}},{\rm{\ }}{{{\rm{z^{\prime}}}}_{149}}} \right) \to \left( {{{{\rm{x^{\prime}}}}_{166}},{\rm{\ }}{{{\rm{y^{\prime}}}}_{166}},{\rm{\ }}{{{\rm{z^{\prime}}}}_{166}}} \right)} \end{array}\end{equation*}$$

Solutions for$\ ( {{\rm{x}}_0^{{\rm{en}}},{\rm{\ y}}_0^{{\rm{en}}},{\rm{\ z}}_0^{{\rm{en}}},{\rm{v}}_{\rm{x}}^{{\rm{en}}},{\rm{v}}_{\rm{y}}^{{\rm{en}}},{\rm{v}}_{\rm{z}}^{{\rm{en}}},{\rm{x}}_0^{{\rm{ex}}},{\rm{\ y}}_0^{{\rm{ex}}},{\rm{\ z}}_0^{{\rm{ex}}},{\rm{v}}_{\rm{x}}^{{\rm{ex}}},{\rm{v}}_{\rm{y}}^{{\rm{ex}}},{\rm{v}}_{\rm{z}}^{{\rm{ex}}}} )$ were obtained as described. Following which $( {{\rm{x}}_1^{\rm{^{\prime}}},{\rm{\ y}}_1^{\rm{^{\prime}}},{\rm{\ z}}_1^{\rm{^{\prime}}}} )$ and $( {{\rm{x}}_{167}^{\rm{^{\prime}}},{\rm{\ y}}_{167}^{\rm{^{\prime}}},{\rm{\ z}}_{167}^{\rm{^{\prime}}}} )$ were determined by extrapolation with base-step vectors respectively (see later discussion in this section).

During model building a small adjustment was permitted to the trajectories of the entering and leaving DNAs. This was carried out (while the nucleosome is aligned at origin) by aligning each segment with the y axis (positive direction) and its base (the point joining the central 129 bp DNA) at origin, followed by ${\rm{\delta }}$ and ${\rm{\varphi }}$ rotations (≤ 5°) with respect to z and x axes, prior to inverse transformation:

$$\begin{equation*}{\left( {{{\rm{C}}_{{\rm{en}}}}} \right)^{ - 1}} \cdot {{\rm{B}}_{{\rm{en}}}} \cdot {{\rm{C}}_{{\rm{en}}}} \cdot \left[ {\begin{array}{@{}*{4}{c}@{}} {{{{\rm{x^{\prime}}}}_1}}&{{{{\rm{x^{\prime}}}}_2}}& \ldots &{{{{\rm{x^{\prime}}}}_{19}}}\\ {{{{\rm{y^{\prime}}}}_1}}&{{{{\rm{y^{\prime}}}}_2}}& \ldots &{{{{\rm{y^{\prime}}}}_{19}}}\\ {{{{\rm{z^{\prime}}}}_1}}&{{{{\rm{z^{\prime}}}}_2}}& \ldots &{{{{\rm{z^{\prime}}}}_{19}}}\\ 1&1& \ldots &1 \end{array}} \right] = \left[ {\begin{array}{@{}*{4}{c}@{}} {{{{\rm{x^{\prime\prime}}}}_1}}&{{{{\rm{x^{\prime\prime}}}}_2}}& \ldots &{{{{\rm{x^{\prime\prime}}}}_{19}}}\\ {{{{\rm{y^{\prime\prime}}}}_1}}&{{{{\rm{y^{\prime\prime}}}}_2}}& \ldots &{{{{\rm{y^{\prime\prime}}}}_{19}}}\\ {{{{\rm{z^{\prime\prime}}}}_1}}&{{{{\rm{z^{\prime\prime}}}}_2}}& \ldots &{{{{\rm{z^{\prime\prime}}}}_{19}}}\\ 1&1& \ldots &1 \end{array}} \right]\end{equation*}$$

$$\begin{equation*}{\left( {{{\rm{C}}_{{\rm{ex}}}}} \right)^{ - 1}} \cdot {{\rm{B}}_{{\rm{ex}}}} \cdot {{\rm{C}}_{{\rm{ex}}}} \cdot \left[ {\begin{array}{@{}*{4}{c}@{}} {{{{\rm{x^{\prime}}}}_{149}}}&{{{{\rm{x^{\prime}}}}_{150}}}& \ldots &{{{{\rm{x^{\prime}}}}_{167}}}\\ {{{{\rm{y^{\prime}}}}_{149}}}&{{{{\rm{y^{\prime}}}}_{150}}}& \ldots &{{{{\rm{y^{\prime}}}}_{167}}}\\ {{{{\rm{z^{\prime}}}}_{149}}}&{{{{\rm{z^{\prime}}}}_{150}}}& \ldots &{{{{\rm{z^{\prime}}}}_{167}}}\\ 1&1& \ldots &1 \end{array}} \right] = \left[ {\begin{array}{@{}*{4}{c}@{}} {{{{\rm{x^{\prime\prime}}}}_{149}}}&{{{{\rm{x^{\prime\prime}}}}_{150}}}& \ldots &{{{{\rm{x^{\prime\prime}}}}_{167}}}\\ {{{{\rm{y^{\prime\prime}}}}_{149}}}&{{{{\rm{y^{\prime\prime}}}}_{150}}}& \ldots &{{{{\rm{y^{\prime\prime}}}}_{167}}}\\ {{{{\rm{z^{\prime\prime}}}}_{149}}}&{{{{\rm{z^{\prime\prime}}}}_{150}}}& \ldots &{{{{\rm{z^{\prime\prime}}}}_{167}}}\\ 1&1& \ldots &1 \end{array}} \right]\end{equation*}$$

where

$$\begin{equation*}\begin{array}{@{}*{4}{c}@{}} {{{\rm{C}}_{{\rm{en}}}} = \left[ {\begin{array}{@{}*{4}{c}@{}} 1&0&0&0\\ 0&{{\rm{cos}}\left( { - {{\rm{\lambda }}_1}} \right)}&{ - {\rm{sin}}\left( { - {{\rm{\lambda }}_1}} \right)}&0\\ 0&{{\rm{sin}}\left( { - {{\rm{\lambda }}_1}} \right)}&{{\rm{cos}}\left( { - {{\rm{\lambda }}_1}} \right)}&0\\ 0&0&0&1 \end{array}} \right] \cdot } {\left[ {\begin{array}{@{}*{4}{c}@{}} {{\rm{cos}}\left( { - {{\rm{\lambda }}_2}} \right)}&{ - {\rm{sin}}\left( { - {{\rm{\lambda }}_2}} \right)}&0&0\\ {{\rm{sin}}\left( { - {{\rm{\lambda }}_2}} \right)}&{{\rm{cos}}\left( { - {{\rm{\lambda }}_2}} \right)}&0&0\\ 0&0&1&0\\ 0&0&0&1 \end{array}} \right] \cdot \left[ {\begin{array}{@{}*{4}{c}@{}} 1&0&0&{ - {{\rm{x}}_{19}}}\\ 0&1&0&{ - {{\rm{y}}_{19}}}\\ 0&0&1&{ - {{\rm{z}}_{19}}}\\ 0&0&0&1 \end{array}} \right]} \end{array}\end{equation*}$$

$$\begin{equation*}\ {{\rm{\lambda }}_1} = {\rm{\ arcsin}}\left( {\frac{{\left| {\left. {{\rm{z}}_1^{\rm{^{\prime}}} - {\rm{z}}_{19}^{\rm{^{\prime}}}} \right|} \right.}}{{\sqrt {{{\left( {{\rm{x}}_1^{\rm{^{\prime}}} - {\rm{x}}_{19}^{\rm{^{\prime}}}} \right)}^2} + {{\left( {{\rm{y}}_1^{\rm{^{\prime}}} - {\rm{y}}_{19}^{\rm{^{\prime}}}} \right)}^2} + {{\left( {{\rm{z}}_1^{\rm{^{\prime}}} - {\rm{z}}_{19}^{\rm{^{\prime}}}} \right)}^2}} }}} \right),\, {{\rm{\lambda }}_2} = {\rm{\ arctan}}\left( {\frac{{\left| {\left. {{\rm{x}}_1^{\rm{^{\prime}}} - {\rm{x}}_{19}^{\rm{^{\prime}}}} \right|} \right.}}{{\left| {\left. {{\rm{y}}_1^{\rm{^{\prime}}} - {\rm{y}}_{19}^{\rm{^{\prime}}}} \right|} \right.}}} \right)\end{equation*}$$

$$\begin{equation*}\begin{array}{@{}*{4}{c}@{}} {{{\rm{B}}_{{\rm{en}}}} = \left[ {\begin{array}{@{}*{4}{c}@{}} 1&0&0&0\\ 0&{{\rm{cos}}\left( { - {\rm{\varphi }}} \right)}&{ - {\rm{sin}}\left( { - {\rm{\varphi }}} \right)}&0\\ 0&{{\rm{sin}}\left( { - {\rm{\varphi }}} \right)}&{{\rm{cos}}\left( { - {\rm{\varphi }}} \right)}&0\\ 0&0&0&1 \end{array}} \right]{\rm{\ }} \cdot } {\left[ {\begin{array}{@{}*{4}{c}@{}} {{\rm{cos}}\left( { - {\rm{\delta }}} \right)}&{ - {\rm{sin}}\left( { - {\rm{\delta }}} \right)}&0&0\\ {{\rm{sin}}\left( { - {\rm{\delta }}} \right)}&{{\rm{cos}}\left( { - {\rm{\delta }}} \right)}&0&0\\ 0&0&1&0\\ 0&0&0&1 \end{array}} \right]} \end{array}\end{equation*}$$

$$\begin{equation*}\begin{array}{@{}*{4}{c}@{}} {{{\rm{C}}_{{\rm{ex}}}} = \left[ {\begin{array}{@{}*{4}{c}@{}} 1&0&0&0\\ 0&{{\rm{cos}}\left( {{{\rm{\sigma }}_1}} \right)}&{ - {\rm{sin}}\left( {{{\rm{\sigma }}_1}} \right)}&0\\ 0&{{\rm{sin}}\left( {{{\rm{\sigma }}_1}} \right)}&{{\rm{cos}}\left( {{{\rm{\sigma }}_1}} \right)}&0\\ 0&0&0&1 \end{array}} \right] \cdot } {\left[ {\begin{array}{@{}*{4}{c}@{}} {{\rm{cos}}\left( {{{\rm{\sigma }}_2}} \right)}&{ - {\rm{sin}}\left( {{{\rm{\sigma }}_2}} \right)}&0&0\\ {{\rm{sin}}\left( {{{\rm{\sigma }}_2}} \right)}&{{\rm{cos}}\left( {{{\rm{\sigma }}_2}} \right)}&0&0\\ 0&0&1&0\\ 0&0&0&1 \end{array}} \right] \cdot \left[ {\begin{array}{@{}*{4}{c}@{}} 1&0&0&{ - {{\rm{x}}_{149}}}\\ 0&1&0&{ - {{\rm{y}}_{149}}}\\ 0&0&1&{ - {{\rm{z}}_{149}}}\\ 0&0&0&1 \end{array}} \right]} \end{array}\end{equation*}$$

$$\begin{equation*}\ {{\rm{\sigma }}_1} = {\rm{\ arcsin}}\left( {\frac{{\left| {\left. {{\rm{z}}_{167}^{\rm{^{\prime}}} - {\rm{z}}_{149}^{\rm{^{\prime}}}} \right|} \right.}}{{\sqrt {{{\left( {{\rm{x}}_{167}^{\rm{^{\prime}}} - {\rm{x}}_{149}^{\rm{^{\prime}}}} \right)}^2} + {{\left( {{\rm{y}}_{167}^{\rm{^{\prime}}} - {\rm{y}}_{149}^{\rm{^{\prime}}}} \right)}^2} + {{\left( {{\rm{z}}_{167}^{\rm{^{\prime}}} - {\rm{z}}_{149}^{\rm{^{\prime}}}} \right)}^2}} }}} \right),\, {{\rm{\sigma }}_2} = {\rm{\ arctan}}\left( {\frac{{\left| {\left. {{\rm{x}}_{167}^{\rm{^{\prime}}} - {\rm{x}}_{149}^{\rm{^{\prime}}}} \right|} \right.}}{{\left| {\left. {{\rm{y}}_{167}^{\rm{^{\prime}}} - {\rm{y}}_{149}^{\rm{^{\prime}}}} \right|} \right.}}} \right)\end{equation*}$$

$$\begin{equation*}{{\rm{B}}_{{\rm{ex}}}} = \left[ {\begin{array}{@{}*{4}{c}@{}} 1&0&0&0\\ 0&{{\rm{cos}}\left( {\rm{\varphi }} \right)}&{ - {\rm{sin}}\left( {\rm{\varphi }} \right)}&0\\ 0&{{\rm{sin}}\left( {\rm{\varphi }} \right)}&{{\rm{cos}}\left( {\rm{\varphi }} \right)}&0\\ 0&0&0&1 \end{array}} \right]{\rm{\ }} \cdot \left[ {\begin{array}{@{}*{4}{c}@{}} {{\rm{cos}}\left( {\rm{\delta }} \right)}&{ - {\rm{sin}}\left( {\rm{\delta }} \right)}&0&0\\ {{\rm{sin}}\left( {\rm{\delta }} \right)}&{{\rm{cos}}\left( {\rm{\delta }} \right)}&0&0\\ 0&0&1&0\\ 0&0&0&1 \end{array}} \right]\end{equation*}$$

Therefore the base pair coordinates (best fit values) for the 167 bp (central 129 bp + 2 × 19 bp) following adjustment were given as:

$$\begin{equation*}\begin{array}{@{}*{4}{c}@{}} {\left[ {\begin{array}{@{}*{4}{c}@{}} {{{{\rm{x^{\prime\prime}}}}_1}}&{{{{\rm{x^{\prime\prime}}}}_2}}& \ldots &{{{{\rm{x^{\prime\prime}}}}_{19}}}\\ {{{{\rm{y^{\prime\prime}}}}_1}}&{{{{\rm{y^{\prime\prime}}}}_2}}& \ldots &{{{{\rm{y^{\prime\prime}}}}_{19}}}\\ {{{{\rm{z^{\prime\prime}}}}_1}}&{{{{\rm{z^{\prime\prime}}}}_2}}& \ldots &{{{{\rm{z^{\prime\prime}}}}_{19}}}\\ 1&1& \ldots &1 \end{array}} \right],\,\left[ {\begin{array}{@{}*{4}{c}@{}} {{{{\rm{x^{\prime}}}}_{20}}}&{{{{\rm{x^{\prime}}}}_{21}}}& \ldots &{{{{\rm{x^{\prime}}}}_{148}}}\\ {{{{\rm{y^{\prime}}}}_{20}}}&{{{{\rm{y^{\prime}}}}_{21}}}& \ldots &{{{{\rm{y^{\prime}}}}_{148}}}\\ {{{{\rm{z^{\prime}}}}_{20}}}&{{{{\rm{z^{\prime}}}}_{21}}}& \ldots &{{{{\rm{z^{\prime}}}}_{148}}}\\ 1&1& \ldots &1 \end{array}} \right],} {\left[ {\begin{array}{@{}*{4}{c}@{}} {{{{\rm{x^{\prime\prime}}}}_{149}}}&{{{{\rm{x^{\prime\prime}}}}_{150}}}& \ldots &{{{{\rm{x^{\prime\prime}}}}_{167}}}\\ {{{{\rm{y^{\prime\prime}}}}_{149}}}&{{{{\rm{y^{\prime\prime}}}}_{150}}}& \ldots &{{{{\rm{y^{\prime\prime}}}}_{167}}}\\ {{{{\rm{z^{\prime\prime}}}}_{149}}}&{{{{\rm{z^{\prime\prime}}}}_{150}}}& \ldots &{{{{\rm{z^{\prime\prime}}}}_{167}}}\\ 1&1& \ldots &1 \end{array}} \right]} \end{array}\end{equation*}$$

Once again, during model building, the lengths of the entering and leaving DNAs were extended where necessary. In which case the extra base pairs added to each of the 19 bp segments were positioned by multiplying the number of base steps (${\rm{u}},{\rm{v}}$) (upper limits of ${\rm{u}}$ and ${\rm{v}}$ depend on NRL) with base-step vectors (that defines the trajectory) respectively:

$$\begin{equation*}\left[ {\begin{array}{@{}*{1}{c}@{}} {\frac{{{{\rm{x}}_1} - {{\rm{x}}_{19}}}}{{18}}}\\ {\frac{{{{\rm{y}}_1} - {{\rm{y}}_{19}}}}{{18}}}\\ {\frac{{{{\rm{z}}_1} - {{\rm{z}}_{19}}}}{{18}}}\\ 1 \end{array}} \right] \cdot {\rm{u}}\left( {{\rm{u}} = {\rm{integer}}} \right),\,\left[ {\begin{array}{@{}*{1}{c}@{}} {\frac{{{{\rm{x}}_{167}} - {{\rm{x}}_{149}}}}{{18}}}\\ {\frac{{{{\rm{y}}_{167}} - {{\rm{y}}_{149}}}}{{18}}}\\ {\frac{{{{\rm{z}}_{167}} - {{\rm{z}}_{149}}}}{{18}}}\\ 1 \end{array}} \right] \cdot {\rm{v}}\left( {{\rm{v}} = {\rm{integer}}} \right)\end{equation*}$$

Assign all base pair coordinates (167 bp + extensions) with new notation:

$$\begin{equation*}\left[ {\begin{array}{@{}*{4}{c}@{}} {{\rm{x}}_1^{{\rm{n}}0}}&{{\rm{x}}_2^{{\rm{n}}0}}& \ldots &{{\rm{x}}_{167 + {\rm{u}} + {\rm{v}}}^{{\rm{n}}0}}\\ {{\rm{y}}_1^{{\rm{n}}0}}&{{\rm{y}}_2^{{\rm{n}}0}}& \ldots &{{\rm{y}}_{167 + {\rm{u}} + {\rm{v}}}^{{\rm{n}}0}}\\ {{\rm{z}}_1^{{\rm{n}}0}}&{{\rm{z}}_2^{{\rm{n}}0}}& \ldots &{{\rm{z}}_{167 + {\rm{u}} + {\rm{v}}}^{{\rm{n}}0}}\\ 1&1& \ldots &1 \end{array}} \right]\end{equation*}$$

Since the nucleosome was realigned at origin (see above), the z and y axes correspond to its superhelical and dyad axes respectively (the dyad axis here is equivalent to 2-fold symmetry axis of the nucleosomal DNA) (Figure 1). The normal to the nucleosomal disc (defined by its bisecting plane) was given as:

$$\begin{equation*} {{{\vec{\rm V}}}_{{\rm{nm}}0}} = \left[ {\begin{array}{@{}*{4}{c}@{}} {{\rm{cos}}\left( { - {\rm{arctan}}\left( {\frac{{{\rm{q}} \cdot {\rm{\pi }}}}{{2{\rm{a}}}}} \right)} \right)}&0&{ - {\rm{sin}}\left( { - {\rm{arctan}}\left( {\frac{{{\rm{q}} \cdot {\rm{\pi }}}}{{2{\rm{a}}}}} \right)} \right)}&0\\ 0&1&0&0\\ {{\rm{sin}}\left( { - {\rm{arctan}}\left( {\frac{{{\rm{q}} \cdot {\rm{\pi }}}}{{2{\rm{a}}}}} \right)} \right)}&0&{{\rm{cos}}\left( { - {\rm{arctan}}\left( {\frac{{{\rm{q}} \cdot {\rm{\pi }}}}{{2{\rm{a}}}}} \right)} \right)}&0\\ 0&0&0&1 \end{array}} \right]{\rm{\ }} \cdot \left[ {\begin{array}{@{}*{1}{c}@{}} 0\\ 0\\ 1\\ 1 \end{array}} \right] \end{equation*}$$

### Model building

Fibre models were constructed by positioning individual nucleosomes as a starting point. The procedures described hereafter refer to the 1-start fibre (4). The 2-start structure was built similarly except the odd and even number nucleosomes of an array reside on two separate helical paths.

**Figure 2.**
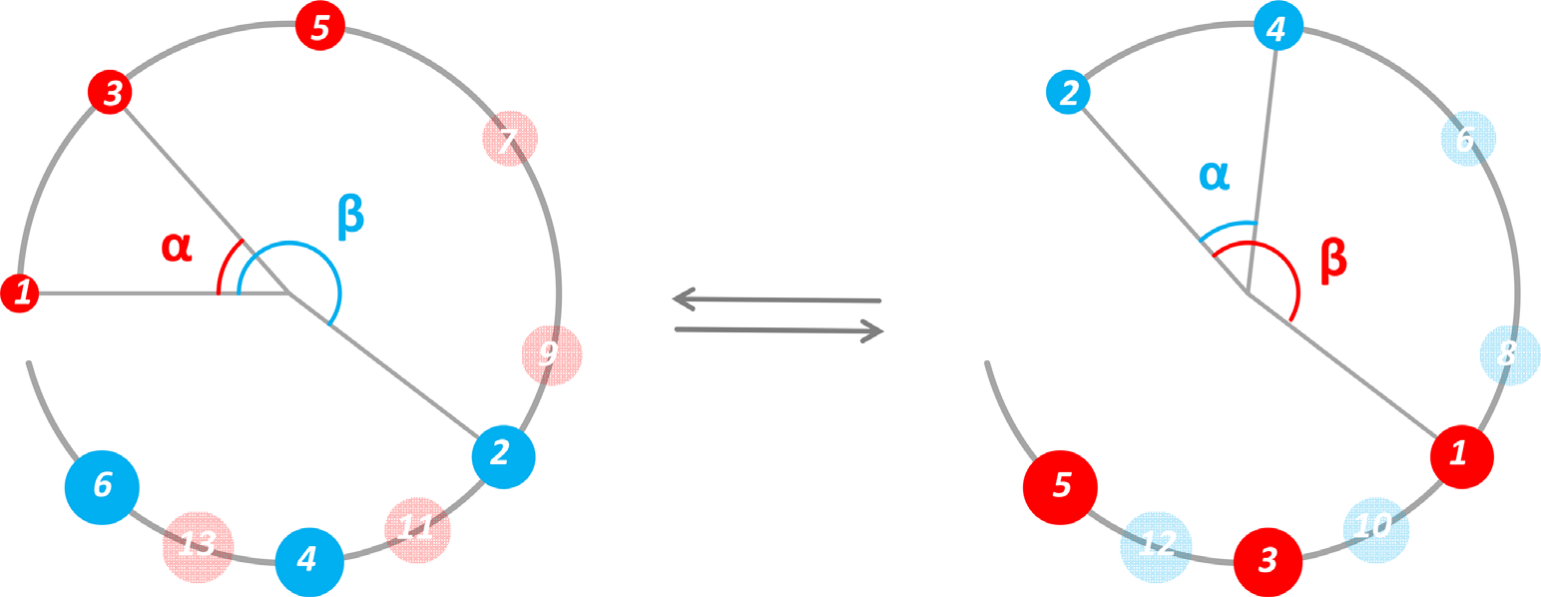
Schematic of the 1-start crossed linker model of the chromatin fibre. The large open circle represents the start of a left-handed helical path rising towards the reader, on which both the odd (red sphere) and even (blue sphere) number nucleosomes reside. The ratio β /α determines the interdigitating pattern of the two series, which, in this example, gives rise to (1 3 5 7 9 ^2^ 11 ^4^ 13 ^6^…) or alternatively (2 4 6 8 ^1^ 10 ^3^ 12 ^5^…) because nucleosomes can interdigitate with either those above or below of the neighbouring stacks. The adjacent nucleosomes in this example are identified as (i±7, i, i±9).

The schematic in Figure 2 shows a helical stack of nucleosomes (left handed), of radius ${\rm{r}}$ and pitch $\ 2{\rm{\pi h}}$, on which red and blue spheres represent odd and even number nucleosomes of an array respectively. The spacing is given by ${\rm{\ \alpha }}$, while the two series start relative to one another by $\ {\rm{\beta }}$. As the two series extend the odd and even number nucleosomes interdigitate (4). Each nucleosome was orientated by having its superhelical axis tangent to the helical path, around which the nucleosomal disc was rotated by${\rm{\ }} \pm {\rm{\theta }}$, thereby specifying the direction of its dyad axis (here equivalent to 2-fold symmetry axis of the nucleosomal DNA).

**Figure 3.**
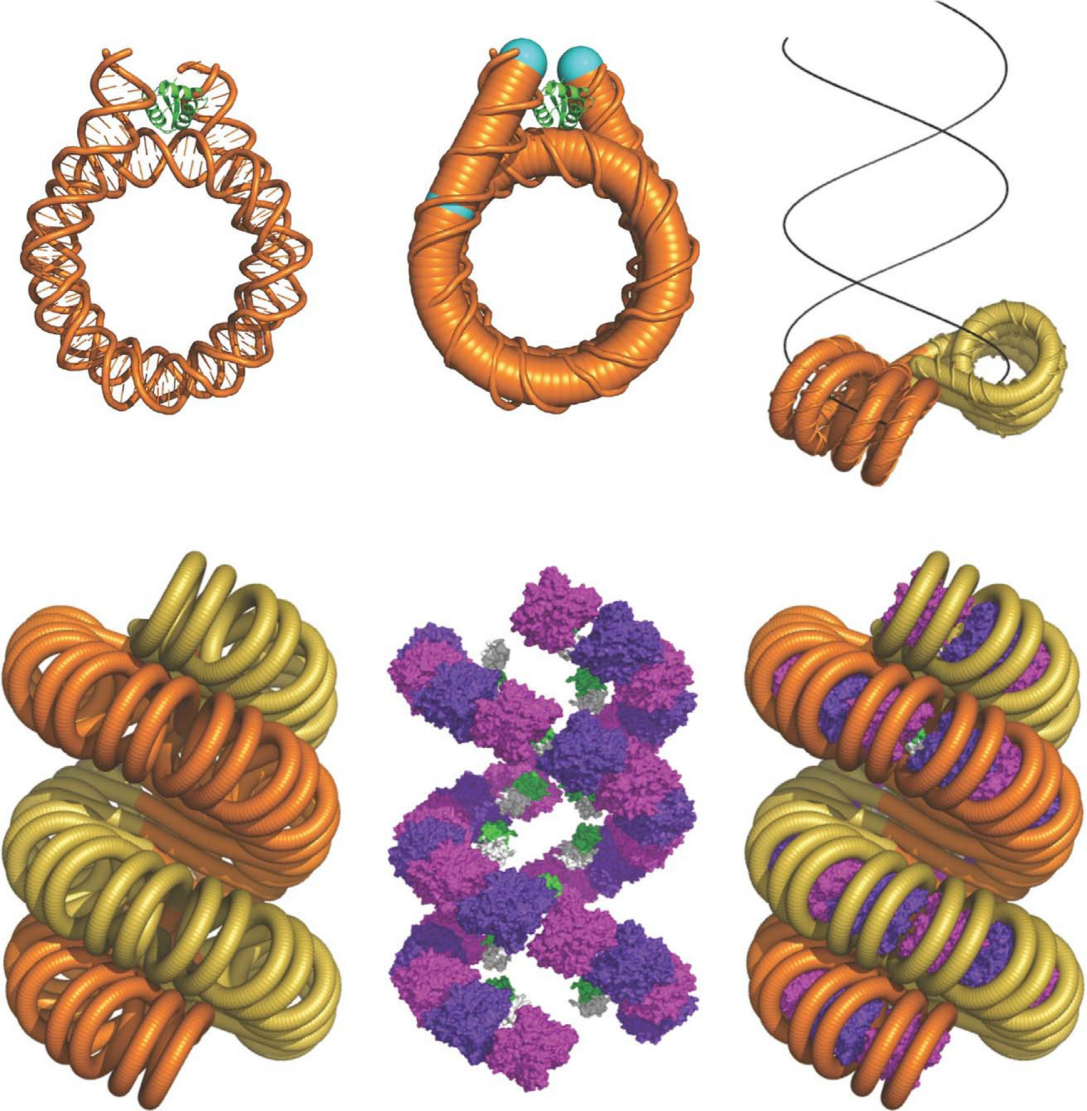
Construction of fibre models. The path of DNA was represented as a smooth tube defined by best fit values of base pair coordinates. The entering and leaving DNAs (the segment between cyan marks) approximate a straight trajectory. The chromatosome structure was used as a geometric unit for model building.

Fibre models were constructed with the DNA path alone for clarity (without the histones). In effect the structure features a continuation of points defined by the best fit base pair coordinates, forming a smooth tube (Figure 3). The first members $( {{\rm{n}}1,{\rm{\ n}}2} )$ of the odd and even number nucleosomes are positioned respectively by:

$$\begin{equation*}\begin{array}{@{}*{4}{c}@{}} {{{\rm{D}}_{{\rm{n}}1}} \cdot \left[ {\begin{array}{@{}*{4}{c}@{}} {{\rm{x}}_1^{{\rm{n}}0}}&{{\rm{x}}_2^{{\rm{n}}0}}& \ldots &{{\rm{x}}_{167 + {\rm{u}} + {\rm{v}}}^{{\rm{n}}0}}\\ {{\rm{y}}_1^{{\rm{n}}0}}&{{\rm{y}}_2^{{\rm{n}}0}}& \ldots &{{\rm{y}}_{167 + {\rm{u}} + {\rm{v}}}^{{\rm{n}}0}}\\ {{\rm{z}}_1^{{\rm{n}}0}}&{{\rm{z}}_2^{{\rm{n}}0}}& \ldots &{{\rm{z}}_{167 + {\rm{u}} + {\rm{v}}}^{{\rm{n}}0}}\\ 1&1& \ldots &1 \end{array}} \right]\,{\rm{and}}\,} {{{\rm{D}}_{{\rm{n}}2}} \cdot \left[ {\begin{array}{@{}*{4}{c}@{}} {{\rm{x}}_1^{{\rm{n}}0}}&{{\rm{x}}_2^{{\rm{n}}0}}& \ldots &{{\rm{x}}_{167 + {\rm{u}} + {\rm{v}}}^{{\rm{n}}0}}\\ {{\rm{y}}_1^{{\rm{n}}0}}&{{\rm{y}}_2^{{\rm{n}}0}}& \ldots &{{\rm{y}}_{167 + {\rm{u}} + {\rm{v}}}^{{\rm{n}}0}}\\ {{\rm{z}}_1^{{\rm{n}}0}}&{{\rm{z}}_2^{{\rm{n}}0}}& \ldots &{{\rm{z}}_{167 + {\rm{u}} + {\rm{v}}}^{{\rm{n}}0}}\\ 1&1& \ldots &1 \end{array}} \right]} \end{array}\end{equation*}$$

where

$$\begin{eqnarray*}{{{\rm{D}}_{{\rm{n}}1}}} &=& \left[ {\begin{array}{@{}*{4}{c}@{}} 1&0&0&{ - {\rm{r}} \cdot {\rm{cos}}\left( 0 \right)}\\ 0&1&0&{{\rm{r}} \cdot {\rm{sin}}\left( 0 \right)}\\ 0&0&1&{{\rm{h}} \cdot 0}\\ 0&0&0&1 \end{array}} \right]{\rm{\ }} \cdot \left[ {\begin{array}{@{}*{4}{c}@{}} {{\rm{cos}}\left( {\frac{{ - {\rm{\pi }}}}{2}} \right)}&{ - {\rm{sin}}\left( {\frac{{ - {\rm{\pi }}}}{2}} \right)}&0&0\\ {{\rm{sin}}\left( {\frac{{ - {\rm{\pi }}}}{2}} \right)}&{{\rm{cos}}\left( {\frac{{ - {\rm{\pi }}}}{2}} \right)}&0&0\\ 0&0&1&0\\ 0&0&0&1 \end{array}} \right] \cdot \left[ {\begin{array}{@{}*{4}{c}@{}} {{\rm{cos}}\left( { - {\rm{arctan}}\left( {\frac{{\rm{h}}}{{\rm{r}}}} \right)} \right)}&0&{ - {\rm{sin}}\left( { - {\rm{arctan}}\left( {\frac{{\rm{h}}}{{\rm{r}}}} \right)} \right)}&0\\ 0&1&0&0\\ {{\rm{sin}}\left( { - {\rm{arctan}}\left( {\frac{{\rm{h}}}{{\rm{r}}}} \right)} \right)}&0&{{\rm{cos}}\left( { - {\rm{arctan}}\left( {\frac{{\rm{h}}}{{\rm{r}}}} \right)} \right)}&0\\ 0&0&0&1 \end{array}} \right] \\ &&\quad\qquad\qquad\qquad\qquad\times \, \left[ {\begin{array}{@{}*{4}{c}@{}} 1&0&0&0\\ 0&{{\rm{cos}}\left( {\rm{\theta }} \right)}&{ - {\rm{sin}}\left( {\rm{\theta }} \right)}&0\\ 0&{{\rm{sin}}\left( {\rm{\theta }} \right)}&{{\rm{cos}}\left( {\rm{\theta }} \right)}&0\\ 0&0&0&1 \end{array}} \right] \cdot {\left[ {\begin{array}{@{}*{4}{c}@{}} {{\rm{cos}}\left( {\frac{{ - {\rm{\pi }}}}{2}} \right)}&0&{ - {\rm{sin}}\left( {\frac{{ - {\rm{\pi }}}}{2}} \right)}&0\\ 0&1&0&0\\ {{\rm{sin}}\left( {\frac{{ - {\rm{\pi }}}}{2}} \right)}&0&{{\rm{cos}}\left( {\frac{{ - {\rm{\pi }}}}{2}} \right)}&0\\ 0&0&0&1 \end{array}} \right]}\end{eqnarray*}$$

$$\begin{eqnarray*} {{{\rm{D}}_{{\rm{n}}2}}} &=& \left[ {\begin{array}{@{}*{4}{c}@{}} 1&0&0&{ - {\rm{r}} \cdot {\rm{cos}}\left( {\rm{\beta }} \right)}\\ 0&1&0&{{\rm{r}} \cdot {\rm{sin}}\left( {\rm{\beta }} \right)}\\ 0&0&1&{{\rm{h}} \cdot {\rm{\beta }}}\\ 0&0&0&1 \end{array}} \right]{\rm{\ }} \cdot \left[ {\begin{array}{@{}*{4}{c}@{}} {{\rm{cos}}\left( {\frac{{3{\rm{\pi }}}}{2} - {\rm{\beta }}} \right)}&{ - {\rm{sin}}\left( {\frac{{3{\rm{\pi }}}}{2} - {\rm{\beta }}} \right)}&0&0\\ {{\rm{sin}}\left( {\frac{{3{\rm{\pi }}}}{2} - {\rm{\beta }}} \right)}&{{\rm{cos}}\left( {\frac{{3{\rm{\pi }}}}{2} - {\rm{\beta }}} \right)}&0&0\\ 0&0&1&0\\ 0&0&0&1 \end{array}} \right] \cdot \left[ {\begin{array}{@{}*{4}{c}@{}} {{\rm{cos}}\left( { - {\rm{arctan}}\left( {\frac{{\rm{h}}}{{\rm{r}}}} \right)} \right)}&0&{ - {\rm{sin}}\left( { - {\rm{arctan}}\left( {\frac{{\rm{h}}}{{\rm{r}}}} \right)} \right)}&0\\ 0&1&0&0\\ {{\rm{sin}}\left( { - {\rm{arctan}}\left( {\frac{{\rm{h}}}{{\rm{r}}}} \right)} \right)}&0&{{\rm{cos}}\left( { - {\rm{arctan}}\left( {\frac{{\rm{h}}}{{\rm{r}}}} \right)} \right)}&0\\ 0&0&0&1 \end{array}} \right] \\ &&\qquad\qquad\qquad\qquad\qquad\times \, {\left[ {\begin{array}{@{}*{4}{c}@{}} 1&0&0&0\\ 0&{{\rm{cos}}\left( { - {\rm{\theta }}} \right)}&{ - {\rm{sin}}\left( { - {\rm{\theta }}} \right)}&0\\ 0&{{\rm{sin}}\left( { - {\rm{\theta }}} \right)}&{{\rm{cos}}\left( { - {\rm{\theta }}} \right)}&0\\ 0&0&0&1 \end{array}} \right] \cdot \left[ {\begin{array}{@{}*{4}{c}@{}} {{\rm{cos}}\left( {\frac{{\rm{\pi }}}{2}} \right)}&0&{ - {\rm{sin}}\left( {\frac{{\rm{\pi }}}{2}} \right)}&0\\ 0&1&0&0\\ {{\rm{sin}}\left( {\frac{{\rm{\pi }}}{2}} \right)}&0&{{\rm{cos}}\left( {\frac{{\rm{\pi }}}{2}} \right)}&0\\ 0&0&0&1 \end{array}} \right]} \end{eqnarray*}$$

Subsequent members of each series relate to the first by $n \cdot \alpha$ rotation and $h \cdot n \cdot \alpha$ translation with respect to the fibre axis (z axis):

$$\begin{equation*}\left[ {\begin{array}{@{}*{4}{c}@{}} {{\rm{cos}}\left( { - {\rm{n}} \cdot {\rm{\alpha }}} \right)}&{ - {\rm{sin}}\left( { - {\rm{n}} \cdot {\rm{\alpha }}} \right)}&0&0\\ {{\rm{sin}}\left( { - {\rm{n}} \cdot {\rm{\alpha }}} \right)}&{{\rm{cos}}\left( { - {\rm{n}} \cdot {\rm{\alpha }}} \right)}&0&0\\ 0&0&1&{{\rm{h}} \cdot {\rm{n}} \cdot {\rm{\alpha }}}\\ 0&0&0&1 \end{array}} \right],\ \left( {{\rm{n}} = 1,{\rm{\ }}2,{\rm{\ }}3 \ldots } \right)\end{equation*}$$

#### Distance between adjacent nucleosomes

The centre to centre distances between a nucleosome and its two adjacent neighbours are given respectively by:

$$\begin{equation*}\sqrt {{{\left( { - {\rm{r}} \cdot {\rm{cos}}\left( {\rm{\beta }} \right) + {\rm{r}} \cdot {\rm{cos}}\left( {{\rm{m}} \cdot {\rm{\alpha }}} \right)} \right)}^2} + {{\left( {{\rm{r}} \cdot {\rm{sin}}\left( {\rm{\beta }} \right) - {\rm{r}} \cdot {\rm{sin}}\left( {{\rm{m}} \cdot {\rm{\alpha }}} \right)} \right)}^2} + {{\left( {{\rm{\beta }} \cdot {\rm{h}} - {\rm{m}} \cdot {\rm{\alpha }} \cdot {\rm{h}}} \right)}^2}} \end{equation*}$$

and

$$\begin{equation*}\sqrt {{{\left( { - {\rm{r}} \cdot {\rm{cos}}\left( {\left( {{\rm{m}} + 1} \right) \cdot {\rm{\alpha }}} \right) + {\rm{r}} \cdot {\rm{cos}}\left( {\rm{\beta }} \right)} \right)}^2} + {{\left( {{\rm{r}} \cdot {\rm{sin}}\left( {{\rm{m}} + 1) \cdot {\rm{\alpha }}} \right) - {\rm{r}} \cdot {\rm{sin}}\left( {\rm{\beta }} \right)} \right)}^2} + {{\left( {\left( {{\rm{m}} + 1} \right) \cdot {\rm{\alpha }} \cdot {\rm{h}} - {\rm{\beta }} \cdot {\rm{h}}} \right)}^2}} \end{equation*}$$

where

$$\begin{equation*}{\rm{m}} = {\rm{int}}\left( {\frac{{\rm{\beta }}}{{\rm{\alpha }}}} \right){\rm{\ }}\left( {{\rm{the\ integer\ part\ of\ }}\frac{{\rm{\beta }}}{{\rm{\alpha }}}} \right)\end{equation*}$$

#### Distance between linker DNAs

Linker DNAs were modelled in a straight trajectory between the end points of the leaving and entering DNAs of consecutive nucleosomes. Their base pair coordinates are determined using a base-step vector as described. With their coordinates known, the path of each linker can be expressed as a line (vector) through a point, for example for two linkers (${\rm{L}}{{\rm{k}}_1},{\rm{\ L}}{{\rm{k}}_2}$) (${\rm{L}}{{\rm{k}}_1}$ refers to the linker between nucleosome ${{\rm{n}}_1}$ and ${{\rm{n}}_2}$):

$$\begin{equation*}\left[ {\begin{array}{@{}*{1}{c}@{}} {{{\rm{x}}_1}}\\ {{{\rm{y}}_1}}\\ {{{\rm{z}}_1}} \end{array}} \right],{\rm{\ }}{{{\vec{\rm V}}}_1} = \left[ {\begin{array}{@{}*{1}{c}@{}} {{{\rm{i}}_1}}\\ {{{\rm{j}}_1}}\\ {{{\rm{k}}_1}} \end{array}} \right]\,{\rm{and}}\,\left[ {\begin{array}{@{}*{1}{c}@{}} {{{\rm{x}}_2}}\\ {{{\rm{y}}_2}}\\ {{{\rm{z}}_2}} \end{array}} \right],{\rm{\ }}{{{\vec{\rm V}}}_2} = \left[ {\begin{array}{@{}*{1}{c}@{}} {{{\rm{i}}_2}}\\ {{{\rm{j}}_2}}\\ {{{\rm{k}}_2}} \end{array}} \right]\end{equation*}$$

The distance between the two lines is given by:

$$\begin{equation*}\sqrt {{{\left( {{{\rm{x}}_2} + {\rm{t}} \cdot {{\rm{i}}_2} - {{\rm{x}}_1} - {\rm{s}} \cdot {{\rm{i}}_1}} \right)}^2} + {{\left( {{{\rm{y}}_2} + {\rm{t}} \cdot {{\rm{j}}_2} - {{\rm{y}}_1} - {\rm{s}} \cdot {{\rm{j}}_1}} \right)}^2} + {{\left( {{{\rm{z}}_2} + {\rm{t}} \cdot {{\rm{k}}_2} - {{\rm{z}}_1} - {\rm{s}} \cdot {{\rm{k}}_1}} \right)}^2}} \end{equation*}$$

where

$$\begin{equation*}{\rm{s}} = \frac{{\det \left[ {\begin{array}{@{}*{2}{c}@{}} { - \left( {{{\rm{x}}_2} - {{\rm{x}}_1}} \right){{\rm{i}}_1} - \left( {{{\rm{y}}_2} - {{\rm{y}}_1}} \right){{\rm{j}}_1} - \left( {{{\rm{z}}_2} - {{\rm{z}}_1}} \right){{\rm{k}}_1}}&{\left( {{{\rm{k}}_2}{{\rm{k}}_1} + {{\rm{j}}_2}{{\rm{j}}_1} + {{\rm{i}}_2}{{\rm{i}}_1}} \right)}\\ { - \left( {{{\rm{x}}_2} - {{\rm{x}}_1}} \right){{\rm{i}}_2} - \left( {{{\rm{y}}_2} - {{\rm{y}}_1}} \right){{\rm{j}}_2} - \left( {{{\rm{z}}_2} - {{\rm{z}}_1}} \right){{\rm{k}}_2}}&{\left( {{{\rm{k}}_2}^2 + {{\rm{j}}_2}^2 + {{\rm{i}}_2}^2} \right)} \end{array}} \right]}}{{\det \left[ {\begin{array}{@{}*{2}{c}@{}} { - \left( {{{\rm{k}}_1}^2 + {{\rm{j}}_1}^2 + {{\rm{i}}_1}^2} \right)}&{({{\rm{k}}_2}{{\rm{k}}_1} + {{\rm{j}}_2}{{\rm{j}}_1} + {{\rm{i}}_2}{{\rm{i}}_1})}\\ { - \left( {{{\rm{k}}_1}{{\rm{k}}_2} + {{\rm{j}}_1}{{\rm{j}}_2} + {{\rm{i}}_1}{{\rm{i}}_2}} \right)}&{\left( {{{\rm{k}}_2}^2 + {{\rm{j}}_2}^2 + {{\rm{i}}_2}^2} \right)} \end{array}} \right]}}\end{equation*}$$

$$\begin{equation*}{\rm{t}} = \frac{{\det \left[ {\begin{array}{@{}*{2}{c}@{}} { - \left( {{{\rm{k}}_1}^2 + {{\rm{j}}_1}^2 + {{\rm{i}}_1}^2} \right)}&{ - \left( {{{\rm{x}}_2} - {{\rm{x}}_1}} \right){{\rm{i}}_1} - \left( {{{\rm{y}}_2} - {{\rm{y}}_1}} \right){{\rm{j}}_1} - \left( {{{\rm{z}}_2} - {{\rm{z}}_1}} \right){{\rm{k}}_1}}\\ { - \left( {{{\rm{k}}_1}{{\rm{k}}_2} + {{\rm{j}}_1}{{\rm{j}}_2} + {{\rm{i}}_1}{{\rm{i}}_2}} \right)}&{ - \left( {{{\rm{x}}_2} - {{\rm{x}}_1}} \right){{\rm{i}}_2} - \left( {{{\rm{y}}_2} - {{\rm{y}}_1}} \right){{\rm{j}}_2} - \left( {{{\rm{z}}_2} - {{\rm{z}}_1}} \right){{\rm{k}}_2}} \end{array}} \right]}}{{\det \left[ {\begin{array}{@{}*{2}{c}@{}} { - \left( {{{\rm{k}}_1}^2 + {{\rm{j}}_1}^2 + {{\rm{i}}_1}^2} \right)}&{\left( {{{\rm{k}}_2}{{\rm{k}}_1} + {{\rm{j}}_2}{{\rm{j}}_1} + {{\rm{i}}_2}{{\rm{i}}_1}} \right)}\\ { - \left( {{{\rm{k}}_1}{{\rm{k}}_2} + {{\rm{j}}_1}{{\rm{j}}_2} + {{\rm{i}}_1}{{\rm{i}}_2}} \right)}&{\left( {{{\rm{k}}_2}^2 + {{\rm{j}}_2}^2 + {{\rm{i}}_2}^2} \right)} \end{array}} \right]}}\end{equation*}$$

For a succession of linkers, the distances between immediate neighbours were calculated between ${\rm{L}}{{\rm{k}}_1}$ and ${\rm{L}}{{\rm{k}}_2}$, ${\rm{L}}{{\rm{k}}_2}$ and ${\rm{L}}{{\rm{k}}_3}$, ${\rm{L}}{{\rm{k}}_1}$ and ${\rm{L}}{{\rm{k}}_3}$, ${\rm{L}}{{\rm{k}}_2}$ and ${\rm{L}}{{\rm{k}}_4}$ occurring at the fibre interior, and additionally between entering and leaving DNAs (between the 19 bp segments) at the interface between adjacent nucleosomes, for example between ${{\rm{n}}_9}$ and ${{\rm{n}}_2}$ if ${\rm{int\ }}( {\frac{{\rm{\beta }}}{{\rm{\alpha }}}} ) = {\rm{\ }}4$, between ${{\rm{n}}_{11}}$ and ${{\rm{n}}_2}$ if ${\rm{int\ }}( {\frac{{\rm{\beta }}}{{\rm{\alpha }}}} ) = {\rm{\ }}5$, between ${{\rm{n}}_{13}}$ and ${{\rm{n}}_2}$ if ${\rm{int\ }}( {\frac{{\rm{\beta }}}{{\rm{\alpha }}}} ) = {\rm{\ }}6$ and so on.

Note that ${\rm{int}}( {\frac{{\rm{\beta }}}{{\rm{\alpha }}}} )$ corresponds, in the fibre structure, the interdigitated pattern (Figure 2) between the odd and even number nucleosomes and thereby identifies the two adjacent nucleosomes (in the fibre structure) with regards to their relative positions (distance) in the nucleosome array.

#### Relative orientation of nucleosomes

**Figure 4.**
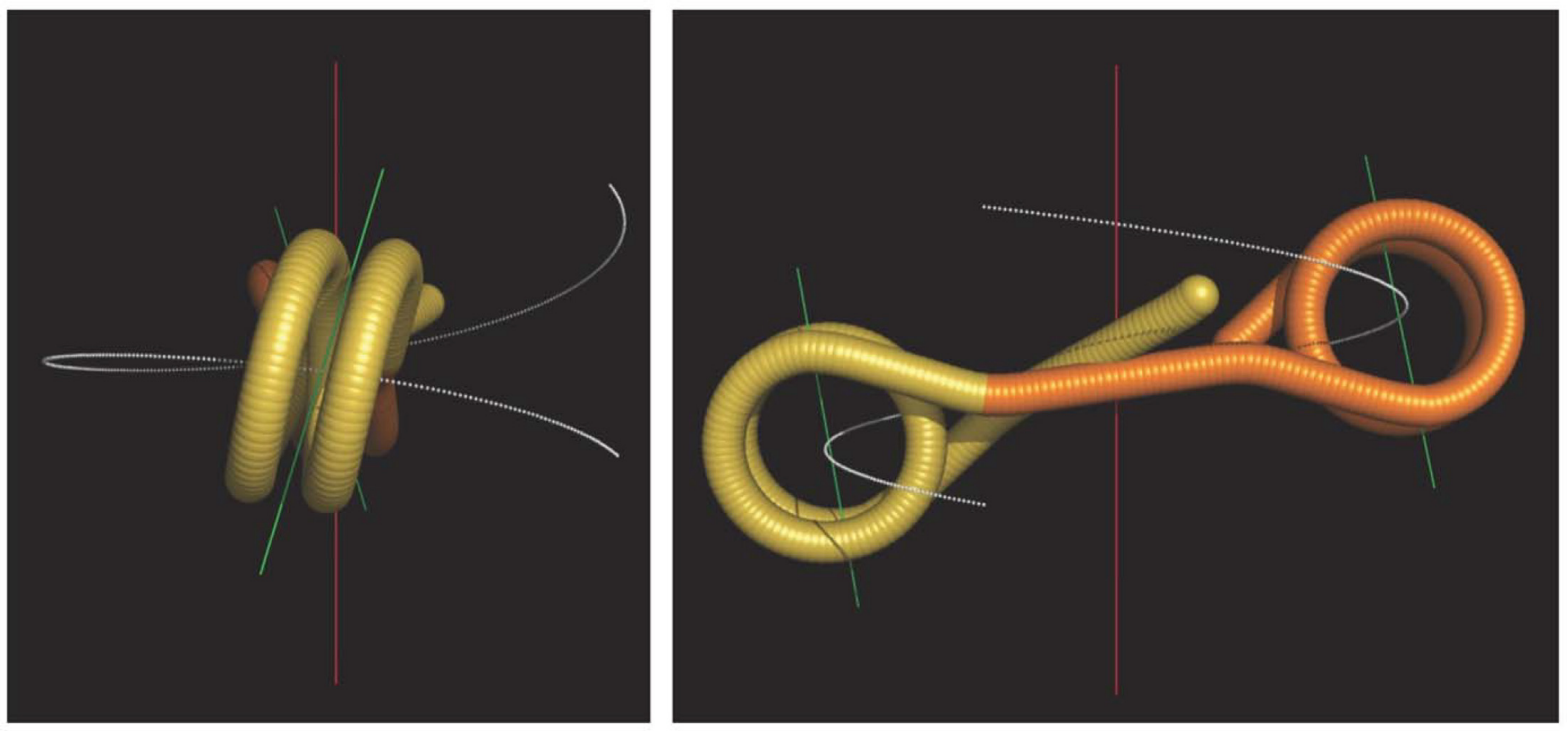
Calculation of relative twist between consecutive nucleosomes. The green lines lie on the bisecting planes of respective nucleosomes, and are orthogonal to the trajectory of the linker DNA. Consequently, their corresponding vectors are cross products between the linker trajectory and the normals to respective nucleosome discs (defined by their bisecting planes). The relative twist between the nucleosomes are given by the angle between the green lines.

The relative twist angles between ${{\rm{n}}_{\rm{i}}}$ and ${{\rm{n}}_{{\rm{i}} + 1}}$ as well as between ${{\rm{n}}_{\rm{i}}}$ and ${{\rm{n}}_{{\rm{i}} - 1}}$ nucleosomes were calculated respectively. For example, the angle between ${{\rm{n}}_1}$ and ${{\rm{n}}_2}$ connected by linker DNA ${\rm{L}}{{\rm{k}}_1}$ is given by:

$$\begin{equation*}{\rm{arccos}}\left( {\frac{{{{{{\vec{\rm V}}}}_{{\rm{b}}1}} \cdot {{{{\vec{\rm V}}}}_{{\rm{b}}2}}}}{{\left| {\left. {{{{{\vec{\rm V}}}}_{{\rm{b}}1}}} \right|} \right.\left| {\left. {{{{{\vec{\rm V}}}}_{{\rm{b}}2}}} \right|} \right.}}} \right)\end{equation*}$$

where ${{{\vec{\rm V}}}_{{\rm{b}}1}}$ and ${{{\vec{\rm V}}}_{{\rm{b}}2}}$ are cross products respectively between the normals (${{{\vec{\rm V}}}_{{\rm{nm}}1}}$, ${{{\vec{\rm V}}}_{{\rm{nm}}2}}$) to the nucleosomal disc (defined by its bisecting plane) and the trajectory of the linker (${{{\vec{\rm V}}}_{{\rm{Lk}}1}}$) (Figure 4), therefore:

$$\begin{equation*}\ {{{\vec{\rm V}}}_{{\rm{b}}1}} = {{{\vec{\rm V}}}_{{\rm{nm}}1}}{\rm{\ }} \times {{{\vec{\rm V}}}_{{\rm{Lk}}1}}\end{equation*}$$

$$\begin{equation*}\ {{{\vec{\rm V}}}_{{\rm{b}}2}} = {{{\vec{\rm V}}}_{{\rm{nm}}2}}{\rm{\ }} \times {{{\vec{\rm V}}}_{{\rm{Lk}}1}}\end{equation*}$$

where

$$\begin{equation*}\ {{{\vec{\rm V}}}_{{\rm{nm}}1}} = \left[ {\begin{array}{@{}*{4}{c}@{}} 1&0&0&0\\ 0&1&0&0\\ 0&0&1&0 \end{array}} \right]{\rm{\ }} \cdot {{\rm{D}}_{{\rm{n}}1}} \cdot {{{\vec{\rm V}}}_{{\rm{nm}}0}}\end{equation*}$$

$$\begin{equation*}{{{\vec{\rm V}}}_{{\rm{nm}}2}} = \left[ {\begin{array}{@{}*{4}{c}@{}} 1&0&0&0\\ 0&1&0&0\\ 0&0&1&0 \end{array}} \right]{\rm{\ }} \cdot {{\rm{D}}_{{\rm{n}}2}} \cdot {{{\vec{\rm V}}}_{{\rm{nm}}0}}\end{equation*}$$

$$\begin{equation*}\ {{{\vec{\rm V}}}_{{\rm{Lk}}1}} = \left[ {\begin{array}{@{}*{4}{c}@{}} 1&0&0&0\\ 0&1&0&0\\ 0&0&1&0 \end{array}} \right]{\rm{\ }} \cdot \left[ {\begin{array}{@{}*{1}{c}@{}} {{\rm{x}}_1^{{\rm{n}}2} - {\rm{x}}_{167 + {\rm{u}} + {\rm{v}}}^{{\rm{n}}1}}\\ {{\rm{y}}_1^{{\rm{n}}2} - {\rm{y}}_{167 + {\rm{u}} + {\rm{v}}}^{{\rm{n}}1}}\\ {{\rm{z}}_1^{{\rm{n}}2} - {\rm{z}}_{167 + {\rm{u}} + {\rm{v}}}^{{\rm{n}}1}}\\ 1 \end{array}} \right]\end{equation*}$$

In addition to above calculation, the twist between consecutive nucleosomes is dictated by the helical twist of the linker DNA. Thus, an alternative calculation (a helical repeat of 10 bp is used in this example, see also RESULTS AND DISCUSSION) based on linker length is given by:

$2{\rm{\pi }}( {1 - {\rm{frac}}( {\frac{{\rm{N}}}{{10}} + 1 - \frac{{51.52}}{{360}}} )} )$, (${\rm{frac}}$ takes the number after the decimal place) where

$$\begin{equation*}{\rm{N}} = \sqrt {{{\left( {{\rm{x}}_{167 + {\rm{u}} + {\rm{v}}}^{\rm n1} - {\rm{x}}_1^{{\rm{n}}2}} \right)}^2} + {{\left( {{\rm{y}}_{167 + {\rm{u}} + {\rm{v}}}^{{\rm{n}}1} - {\rm{y}}_1^{{\rm{n}}2}} \right)}^2} + {{\left( {{\rm{z}}_{167 + {\rm{u}} + {\rm{v}}}^{{\rm{n}}1} - {\rm{z}}_1^{{\rm{n}}2}} \right)}^2}} {\rm{\ }} \cdot \frac{1}{{3.405}} + 19 + {\rm{u}} + {\rm{v}}\end{equation*}$$

${\rm{N}}$ is the number of base pairs of the linker DNA between ${{\rm{n}}_1}$ and ${{\rm{n}}_2}$ core DNA (147 bp), assuming a base-step rise of 3.405 Å. 51.52 (°) is the baseline value of twist with zero linker length, estimated empirically by connecting two core particles in Pymol.

Where the two calculations resulting in equality, then the relative twist between consecutive nucleosomes intrinsic to the structure is consistent with the length of the linker DNA between them. In this situation, the fibre structure should in principle be energetically favourable.

#### Fibre packing density

Fibre packing density expressed as the number of bp per unit volume (Å)^3^ is given by:

$$\begin{equation*}\frac{{\frac{1}{{3.405}}\left( {\begin{array}{@{}*{1}{c}@{}} {\sqrt {{{\left( {{\rm{x}}_{167 + {\rm{u}} + {\rm{v}}}^{{\rm{n}}1} - {\rm{x}}_1^{{\rm{n}}2}} \right)}^2} + {{\left( {{\rm{y}}_{167 + {\rm{u}} + {\rm{v}}}^{{\rm{n}}1} - {\rm{y}}_1^{{\rm{n}}2}} \right)}^2} + {{\left( {{\rm{z}}_{167 + {\rm{u}} + {\rm{v}}}^{{\rm{n}}1} - {\rm{z}}_1^{{\rm{n}}2}} \right)}^2}} }\\ { + \sqrt {{{\left( {{\rm{x}}_{167 + {\rm{u}} + {\rm{v}}}^{{\rm{n}}2} - {\rm{x}}_1^{{\rm{n}}3}} \right)}^2} + {{\left( {{\rm{y}}_{167 + {\rm{u}} + {\rm{v}}}^{{\rm{n}}2} - {\rm{y}}_1^{{\rm{n}}3}} \right)}^2} + {{\left( {{\rm{z}}_{167 + {\rm{u}} + {\rm{v}}}^{{\rm{n}}2} - {\rm{z}}_1^{{\rm{n}}3}} \right)}^2}} } \end{array}} \right) + 2\left( {167 + {\rm{u}} + {\rm{v}} - 1} \right)}}{{{\rm{\alpha }} \cdot {\rm{h}} \cdot {\rm{\pi }}{{\left( {{\rm{r}} + 55} \right)}^2}}}\end{equation*}$$

where 55 (Å) is the radius of the nucleosomal disc.

### Optimisation of geometric variables for fibre packing

Denote:

${{\rm{D}}_{{\rm{fibre}}}}$ – fibre packing density
${{\rm{d}}_{{\rm{gyre}}}}$ – distance between successive gyres of nucleosome stacks ( $ = {\rm{\ }}2{\rm{\pi h}}$)
${{\rm{d}}_{{\rm{n}} - {\rm{n}}}}$ – distance between adjacent nucleosomes (centre to centre)
${{\rm{d}}_{{\rm{Lk}} - {\rm{Lk}}}}$ – distance between linkers
${{\rm{d}}_{{\rm{Lk}}/{\rm{Lk}}}}$ – distance between the entering and leaving linkers (19 bp segment) at nucleosome interfaces
${\rm Tw}_{\rm n - \rm n}^{\rm str}$ – relative twist between consecutive nucleosomes calculated from the fibre geometry (intrinsic to the structure)
${\rm Tw}_{\rm n - \rm n}^{\rm LL}$– relative twist between consecutive nucleosomes calculated according to linker length

To optimize for fibre packing Solver was set up as follows:

iterate $( {{\rm{r}},{\rm{\ h}},{\rm{\ \alpha }},{\rm{\ \beta }},{\rm{\ \theta }}} )$, $( {{\rm{\delta }},{\rm{\ \varphi }}} )$, $( {{\rm{u}},{\rm{\ v}}} )$ for maximal $\frac{{{{\rm{D}}_{{\rm{fibre}}}}}}{{( {{{\rm{d}}_{{\rm{n}}1 - {\rm{n}}2}} + {{\rm{d}}_{{\rm{n}}2 - {\rm{n}}3}}} )}}$

with the following constraints:

$$\begin{equation*}{{\rm{d}}_{{\rm{gyre}}}} \ge 110\end{equation*}$$

$$\begin{equation*}\frac{{11{\rm{\pi }}}}{{180}} \le {\rm{\theta }} \le \frac{{\rm{\pi }}}{{12}}, {{\rm{d}}_{{\rm{n}}1 - {\rm{n}}2}} \ge 57.6, {{\rm{d}}_{{\rm{n}}2 - {\rm{n}}3}} \ge 57.6, 0 \le {\rm{\delta }} \le \frac{{\rm{\pi }}}{{36}}, 0 \le {\rm{\varphi }} \le \frac{{\rm{\pi }}}{{36}}\end{equation*}$$

$$\begin{equation*}{{\rm{d}}_{{\rm{Lk}}1 - {\rm{Lk}}2}} \ge 20, {{\rm{d}}_{{\rm{Lk}}2 - {\rm{Lk}}3}} \ge 20, {{\rm{d}}_{{\rm{Lk}}1 - {\rm{Lk}}3}} \ge 20, {{\rm{d}}_{{\rm{Lk}}2 - {\rm{Lk}}4}} \ge 20 \end{equation*}$$

$$\begin{equation*} {{\rm{d}}_{{\rm{Lk}}9/{\rm{Lk}}2}} \ge 20{\rm{\ if\ int\ }}\left( {\frac{{\rm{\beta }}}{{\rm{\alpha }}}} \right) = {\rm{\ }}4, {{\rm{d}}_{{\rm{Lk}}11/{\rm{Lk}}2}} \ge 20{\rm{\ if\ int\ }}\left( {\frac{{\rm{\beta }}}{{\rm{\alpha }}}} \right) = {\rm{\ }}5, {{\rm{d}}_{{\rm{Lk}}13/{\rm{Lk}}2}} \ge 20{\rm{\ if\ int\ }}\left( {\frac{{\rm{\beta }}}{{\rm{\alpha }}}} \right) = {\rm{\ }}6 \end{equation*}$$

$$\begin{equation*}\ {\rm{Tw}}_{{\rm{n}}1 - {\rm{n}}2}^{{\rm{str}}} = {\rm{Tw}}_{{\rm{n}}1 - {\rm{n}}2}^{{\rm{LL}}}\ , {\rm{Tw}}_{{\rm{n}}2 - {\rm{n}}3}^{{\rm{str}}} = {\rm{Tw}}_{{\rm{n}}2 - {\rm{n}}3}^{{\rm{LL}}}\end{equation*}$$

In maximizing the value of $\frac{{{{\rm{D}}_{{\rm{fibre}}}}}}{{( {{{\rm{d}}_{{\rm{n}}1 - {\rm{n}}2}} + {{\rm{d}}_{{\rm{n}}2 - {\rm{n}}3}}} )}}$ the stacking distance (the denominator) is kept small necessarily (tending toward 57.6 ${\mathring{\rm{A}}}$). The fibre packing density (the numerator) is expressed as the amount of DNA (in terms of bp) per unit fibre volume because Solver solutions based on calculating the number of nucleosomes per 11 nm fibre length could generate suboptimal structures such as having a large central cavity. Although we present our results in nucleosomes/11 nm, since this notation is widely used.

The iteration proceeds from an input of initial values (arbitrary) that give rise to fibres at diameters attainable within the permitted NRL range (∼177–237 bp), over which unique solutions are obtained as local maxima (of packing density) with respective NRLs distributed discontinuously as ‘islands of stability’, corresponding to individual predicted fibres.

Although in principle the initial values are arbitrary, nucleosome arrays in a zigzag configuration arranged to form 1- or 2-start stacks imposes strong geometric constraints. Consequently the workable range of initial values are limited, the iteration converges relatively promptly.

Importantly the resultant structures accord with the necessary specifications. For example, the relative rotation between adjacent nucleosomes is specified within an optimal range (based on published data) such that the helical stack built in this way comprises, effectively, the structural motif of two closely stacked nucleosomes as in the crystal structure (10). In addition, the relative rotation superimposes two complementary histone octamer regions at the interface between the di-nucleosome motifs, facilitating potentially an extensive hydrogen and ionic bond network involving H2A-(A53, E56, E64), H2B-(Q44, V45, E110) with H3-(E73, Q76, D77), H4-(K20, L22, R23) (32).

Note that ($\frac{{11{\rm{\pi }}}}{{180}} \le {\rm{\theta }} \le \frac{{\rm{\pi }}}{{12}}$) means that the relative rotations between adjacent nucleosomes in the fibre were restricted between 22° and 30°, and that (${{\rm{d}}_{{\rm{n}}1 - {\rm{n}}2}} \ge 57.6$) and (${{\rm{d}}_{{\rm{n}}2 - {\rm{n}}3}} \ge 57.6$) indicate that the centre to centre distances between adjacent nucleosomes be ≥57.6 Å (10,32).

The optimization process is animated in movie S2 (Supplementary Data, the symbol ${\rm{\ \psi }}$ in the movie is equivalent to ${\rm{\varphi }}$ in the text, the unit is in degrees) where the fibre structure corresponds to changes in geometric parameters, of which the iteration stops when maximal fibre packing is reached. Most importantly the algorithm refines the relative positions of chromatosome units as well as the length and trajectory of their entering and leaving DNAs, and in turn the linker path. This is therefore a top down model. In contrast, the standard 2-angle model calculation imposes the linker as a tangent line connecting two nucleosome discs, such uniformity limits the variability of feasible structures.

### Calculation of writhe of fibre DNA

The writhing number of fibre DNA was computed using the method formulated elegantly by Levitt (21). Here we summarize the derivations (the letters denoted here accords with Levitt (21) and are independent of preceding formulae).

**Figure 5.**
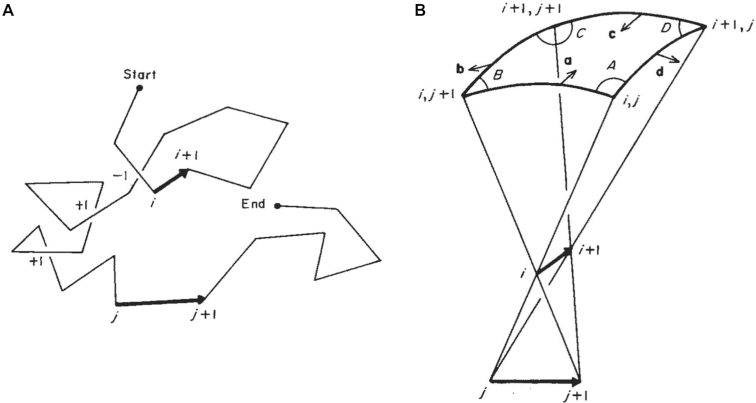
Diagram adapted from Levitt (21) - computation of writhing number from a polygonal curve. (**A**) A polygonal curve defined by a series of vertices where the crossovers between individual segments are indicated with (+1) or (-1) signs (depending on handedness). (**B**) The solid angle (enclosed by four planes) passing through the 2D sphere within which the segments i and j can be seen to cross over. a, b, c, d are normal vectors respectively to the four planes. A, B, C, D are angles between adjacent planes.

Classical calculation of writhing number takes a planar projection of a space curve, followed by summation of the number of (+1) and (–1) crossovers (Figure 5). Results from individual projections of all angles (${\rm{\omega }}$) are then averaged giving the expression:

$$\begin{equation*}{\rm{W\ }} = \frac{1}{{4{\rm{\pi }}}}\ \mathop \int \nolimits {\rm{W}}\left( {\rm{\omega }} \right){\rm{d\omega }}\end{equation*}$$

For a polygonal curve containing a series of vertices:

$$\begin{equation*} {\rm{W}} = \frac{1}{{4{\rm{\pi }}}}\ \mathop \int \nolimits {\rm{W}}\left( {\rm{\omega }} \right){\rm{d\omega \ }} = \frac{1}{{4{\rm{\pi }}}}\ \mathop \int \nolimits \mathop \sum \limits_{{\rm{i}},{\rm{j}} > {\rm{i}}}^{\rm{n}} {{\rm{\delta }}_{{\rm{ij}}}}\left( {\rm{\omega }} \right){\rm{d\omega }} \end{equation*}$$

$$\begin{equation*}{\rm{W\ }} = \mathop \sum \limits_{{\rm{i}},{\rm{j}} > {\rm{i}}}^{\rm{n}} \frac{1}{{4{\rm{\pi }}}}\mathop \int \nolimits{{\rm{\delta }}_{{\rm{ij}}}}\ \left( {\rm{\omega }} \right){\rm{d\omega \ }} = \mathop \sum \limits_{{\rm{i}},{\rm{j}} > {\rm{i}}}^{\rm{n}} {{\rm{W}}_{{\rm{ij}}}}\ \end{equation*}$$

where ${{\rm{W}}_{{\rm{ij}}}}$ is the fraction of projection angles from which crossover can be seen between segments ${\rm{i}}$ and ${\rm{j}}$. ${{\rm{W}}_{{\rm{ij}}}}$ can be obtained by calculating the solid angle ${{\rm{\Omega }}_{{\rm{ij}}}}$ shown in Figure 5 where

$$\begin{equation*}{{\rm{\Omega }}_{{\rm{ij}}}} = {\rm{\ A}} + {\rm{B}} + {\rm{C}} + {\rm{D}} - 2{\rm{\pi }}\end{equation*}$$

The same crossover can be seen from the opposite direction therefore:

$$\begin{equation*}{{\rm{W}}_{{\rm{ij}}}} = \frac{{2{{\rm{\Omega }}_{{\rm{ij}}}}}}{{4{\rm{\pi }}}}\ \end{equation*}$$

Knowing the spatial coordinates of segments ${\rm{\ i}}$ and ${\rm{j}}$, ${\rm{A}}$, ${\rm{B}}$, ${\rm{C}}$, ${\rm{D}}$ can be calculated:

$$\begin{equation*} {{\rm{A}} = {\rm{arccos}}\left( {\frac{{{\rm{a}} \cdot {\rm{d}}}}{{\left| {\left. {\rm{a}} \right|} \right.\left| {\left. {\rm{d}} \right|} \right.}}} \right),\,{\rm{B}} = {\rm{arccos}}\left( {\frac{{{\rm{b}} \cdot {\rm{a}}}}{{\left| {\left. {\rm{b}} \right|} \right.\left| {\left. {\rm{a}} \right|} \right.}}} \right),} {{\rm{C}} = {\rm{arccos}}\left( {\frac{{{\rm{c}} \cdot {\rm{b}}}}{{\left| {\left. {\rm{c}} \right|} \right.\left| {\left. {\rm{b}} \right|} \right.}}} \right),\,{\rm{D}} = {\rm{arccos}}\left( {\frac{{{\rm{d}} \cdot {\rm{c}}}}{{\left| {\left. {\rm{d}} \right|} \right.\left| {\left. {\rm{c}} \right|} \right.}}} \right)} \end{equation*}$$

where

$$\begin{eqnarray*} {\rm{a}} &=& \left( {{{\rm{r}}_{\rm{i}}} - {{\rm{r}}_{{\rm{j}} + 1}}} \right){\rm{\ }} \times \left( {{{\rm{r}}_{\rm{i}}} - {{\rm{r}}_{\rm{j}}}} \right), {\rm{b\ }} = \left( {{{\rm{r}}_{\rm{i}}} - {{\rm{r}}_{{\rm{j}} + 1}}} \right){\rm{\ }} \times \left( {{{\rm{r}}_{{\rm{i}} + 1}} - {{\rm{r}}_{{\rm{j}} + 1}}} \right) \\ {\rm{c}} &=& \left( {{{\rm{r}}_{{\rm{i}} + 1}} - {{\rm{r}}_{\rm{j}}}} \right){\rm{\ }} \times \left( {{{\rm{r}}_{{\rm{i}} + 1}} - {{\rm{r}}_{{\rm{j}} + 1}}} \right), {\rm{d\ }} = \left( {{{\rm{r}}_{{\rm{i}} + 1}} - {{\rm{r}}_{\rm{j}}}} \right){\rm{\ }} \times \left( {{{\rm{r}}_{\rm{i}}} - {{\rm{r}}_{\rm{j}}}} \right) \end{eqnarray*}$$

The sign of ${{\rm{W}}_{{\rm{ij}}}}$ is the same as the vector triple product:

$$\begin{equation*}- \left[ {\left( {{{\rm{r}}_{{\rm{i}} + 1}} - {{\rm{r}}_{\rm{i}}}} \right) \times \left( {{{\rm{r}}_{{\rm{j}} + 1}} - {{\rm{r}}_{\rm{j}}}} \right)} \right] \cdot \left( {{{\rm{r}}_{\rm{i}}} - {{\rm{r}}_{\rm{j}}}} \right)\end{equation*}$$

**Figure 6.**
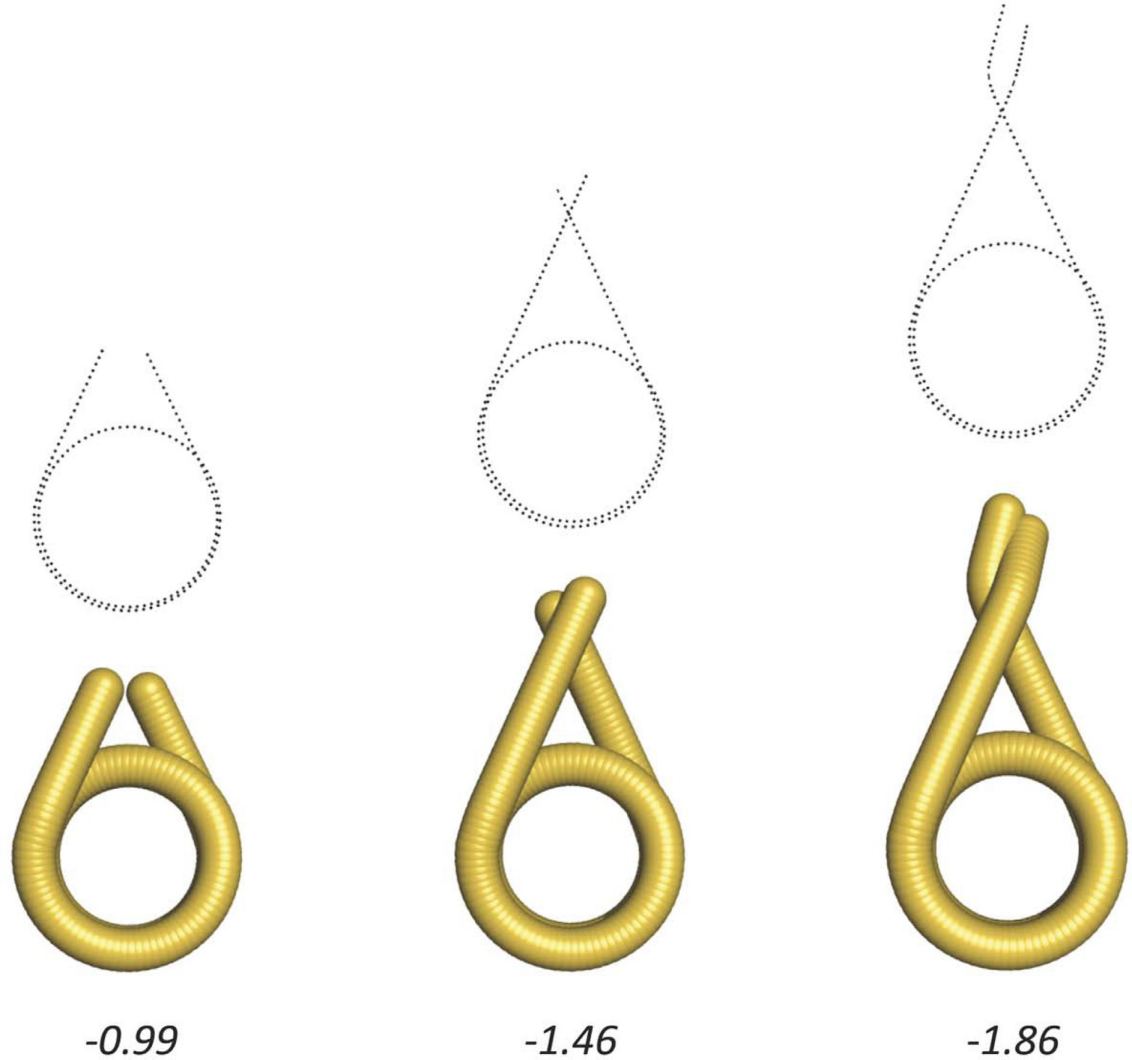
The writhing number of nucleosomal DNA. The DNA paths of three chromatosomes were defined by a series of base pair coordinates shown in black dotted lines. The length of DNA from left to right are 167, 195 and 215 bp, respectively. The writhe values are indicated.

Writhing numbers of fibre DNA were calculated using the DNA path of the 32-mer models. Each data file comprises ∼7000 (depending on NRL) sets of Cartesian coordinates (x, y, z) over the entirety of base steps. Figure 6 illustrates short examples of chromatosomal DNA of varying lengths, for which the writhe values were calculated. We found that a revised method developed by Agarwal *et al.* (23) that achieved a faster computation produced near identical results. However, the formulation by Levitt (21) offers a more direct comparison with past literature.

The linking number of fibre DNA was obtained by subtracting from the writhing number the change in the helical repeat of the linker DNA (NRL minus 147 bp), assuming the intrinsic twist of the nucleosomal DNA is invariant.

## RESULTS AND DISCUSSION

### Chromatin fibres can adopt both the 2-start and 1-start organisations

In this study we construct fibre models using a selection of defining variables $( {{\rm{r}},{\rm{\ h}},{\rm{\ \alpha }},{\rm{\ \beta }},{\rm{\ \theta }},{\rm{\ \delta }},{\rm{\ \varphi }},{\rm{\ u}},{\rm{\ v}}} )$ and seek their optimal values such that the packing of DNA attains the highest possible compaction. Adjacent nucleosomes are configured having two stacking modes alternating based on the crystal and cryo-EM structures (10,12). To minimise torsional stress, we formulate the relative twist of consecutive nucleosomes be agreeable with the length of linker DNA.

Effectively, variables $( {{\rm{r}},{\rm{\ h}},{\rm{\ \alpha }},{\rm{\ \beta }},{\rm{\ \theta }}} )$ determine the diameter and pitch angle of the helical stacks, and on which the spacing and orientation of nucleosomes while the length and trajectory of the entering and leaving DNAs are controlled by $( {{\rm{\delta }},{\rm{\ \varphi }},{\rm{\ u}},{\rm{\ v}}} )$. For both the 2-start and 1-start models the devised procedure produced multiple but markedly discrete solutions (each providing a set of resolved values for individual variables), corresponding to fibres with differing diameters. We infer that this outcome is a consequence of the geometric and topological properties of the crossed-linker model.

**Figure 7.**
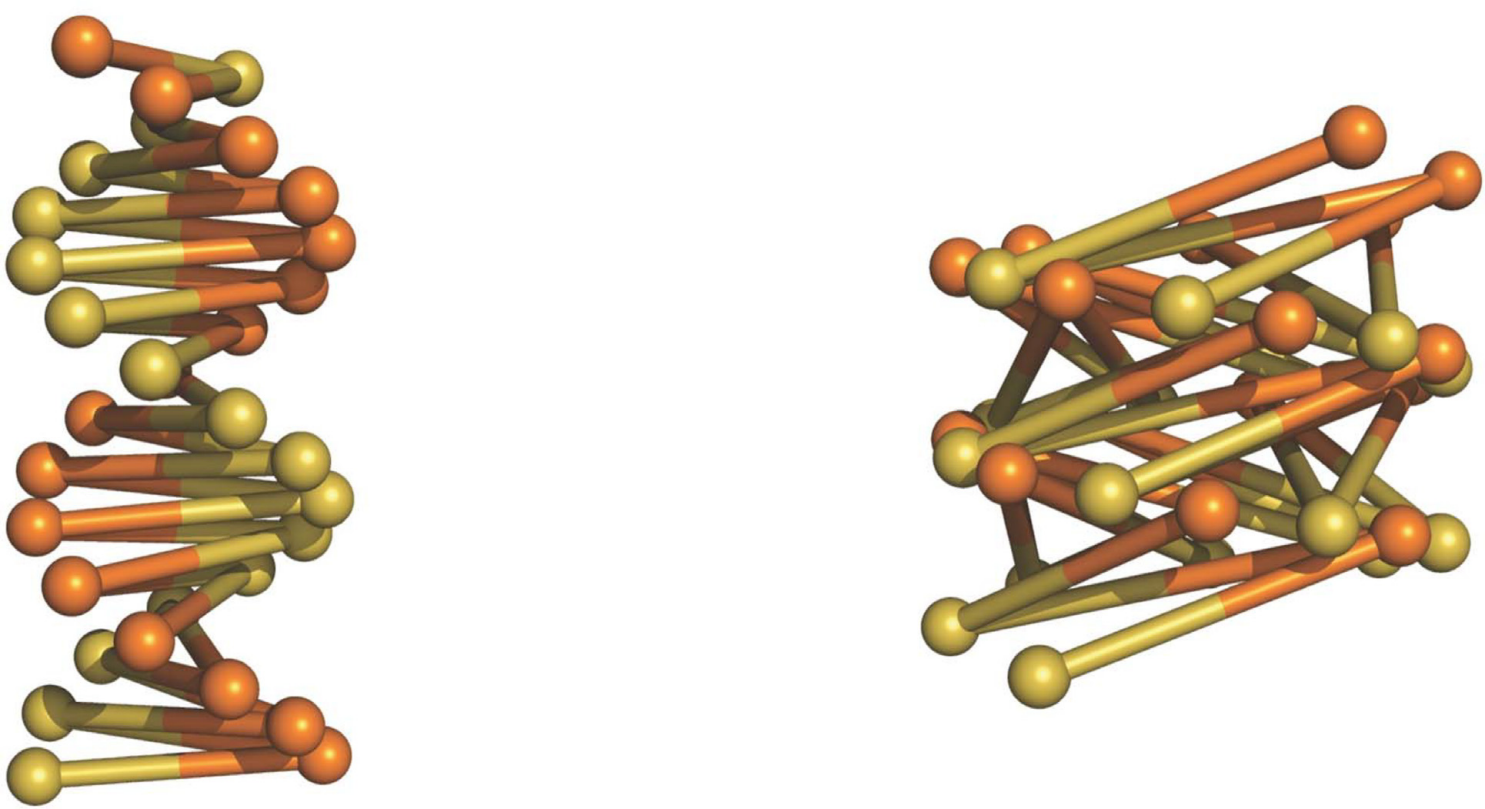
The topology of 2-start and 1-start fibres is analogous to B- and A-form DNA.

During the construction of physical models (not *in silico*), we found that with increased writhing the 2-start fibre can adopt the 1-start configuration through merger of two nucleosome stacks, akin to the conversion of B- to A-form DNA. In the latter structure linker DNAs become more inclined relative to the fibre axis, as is seen with the inclination of base pairs in A-form DNA (Figure 7).

Should the curvature of nucleosome stacks be kept at a constant optimal value, fibres of a smaller diameter would have a high helical pitch, for curvature as a function of radius ($r$) and pitch ($h$) is given by $\ \frac{r}{{{r^2} + {h^2}}}$. Stacked nucleosomes seen in the tetranucleosome and the cryo-EM structure of the chromatin fibre (10,12) form a small curvature and therefore consistent with the 2-start model, in which two nucleosome stacks adopt a high pitch double helix. However, we found in physical models that the 2-start fibre becomes mechanically less stable with increased fibre diameter (with longer linkers), consistent with energetic predictions based on the 2-start configuration (33).

**Figure 8.**
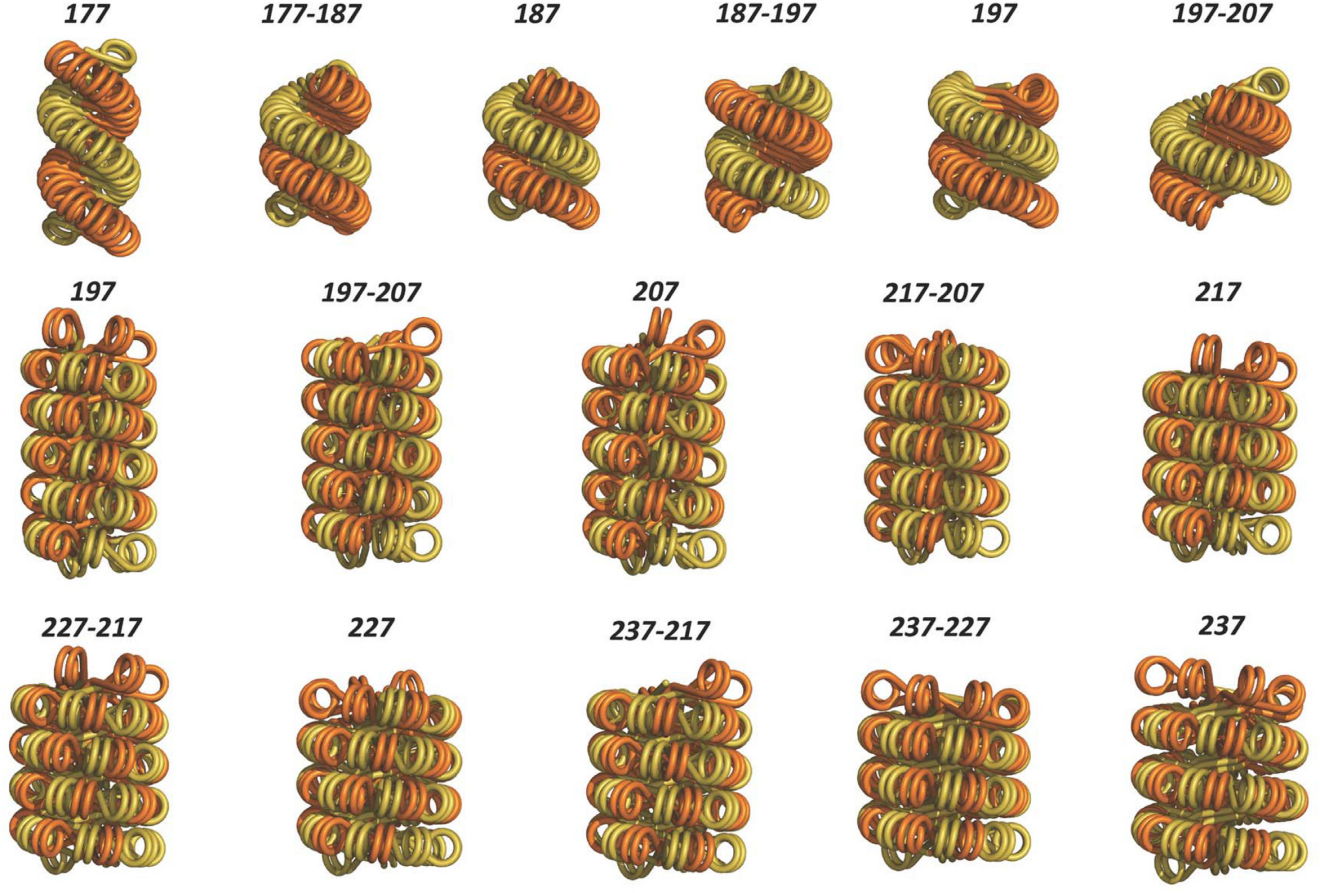
Predicted chromatin fibres with increasing linker length. Chromatin fibres were built based on both the 2-start and 1-start models over NRLs between ∼177 and ∼237 bp. The number above each structure indicates NRL, which can be uniform or between two alternating lengths.

With these considerations, we constructed fibres based on the 2-start model for NRLs ≤197 bp, and ≥197 bp of the 1-start model in which the stability is further provided by inter-locking of the merged stacks (4). In addition, the fibre diameter is now sufficiently large to allow low pitch fibres. Geometric parameters of predicted fibres formed between ∼177 and ∼237 bp NRLs are summarized in Table 1, with the corresponding structures shown in Figure 8 (note that NRLs in Table 1 and Figure 8 are rounded (either up or down) to integer numbers of the calculated NRLs). We found that both the 2- and 1-start models can be built with non-uniform but alternating linkers differing by ∼10 or ∼20 bp. This facility indicates a capacity for accommodating variable linker lengths.

**Table 1.** Variation of fibre parameters for different uniform NRLs and for alternating NRLs.

NRL (bp)	linker (bp)	diameter (nm)	nuc /11 nm	connectivity	model	Wr/nuc	L/nuc
(-177-)_32_	29.14	28.6	7.0	i ± 2	2-start	−1.580	−1.441
(-177–187-)_16_	34.26	30.7	9.2	i ± 2	2-start	−1.594	−1.431
(-187-)_32_	39.68	32.2	10.3	i ± 2	2-start	−1.616	−1.428
(-187–197-)_16_	44.29	34.8	11.2	i ± 2	2-start	−1.611	−1.400
(-197-)_32_	49.75	35.7	10.7	i ± 2	2-start	−1.608	−1.371
(-197–207-)_16_	54.33	38.6	12.4	i ± 2	2-start	−1.619	−1.360
(-197-)_32_	48.87	37.4	10.8	i ± 7, i ± 9	1-start	−1.687	−1.454
(-197–207-)_16_	54.28	38.4	11.5	i ± 7, i ± 9	1-start	−1.705	−1.446
(-207-)_32_	58.86	39.5	11.0	i ± 7, i ± 9	1-start	−1.683	−1.403
(-217–207-)_16_	64.51	39.7	12.0	i ± 7, i ± 9	1-start	−1.706	−1.399
(-217-)_32_	69.35	42.1	13.6	i ± 9, i ± 11	1-start	−1.693	−1.363
(-227–217-)_16_	75.14	43.3	13.2	i ± 7, i ± 9	1-start	−1.767	−1.409
(-227-)_32_	79.90	45.0	14.5	i ± 9, i ± 11	1-start	−1.747	−1.367
(-237–227-)_16_	85.17	47.1	15.2	i ± 9, i ± 11	1-start	−1.765	−1.359
(-237–217-)_16_	80.28	45.0	13.6	i ± 7, i ± 9	1-start	−1.767	−1.385
(-237-)_32_	90.26	48.1	13.4	i ± 9, i ± 11	1-start	−1.779	−1.349

### Quantization of linker DNA

There have been many suggestions that linker lengths are quantized *in vivo*. An early investigation of oligonucleosomal DNAs (34), and more recently, the genome wide analysis of nucleosome positional signals in *S. cerevisiae* (35,36) showed that linkers in yeast accord with 10 × n + 5 bp (integer n). The same periodicity was seen in nucleosome dimers (two chromatosomes plus linker) of rat liver chromatin (37), but an analogous study of human K562 cells revealed the rule of 10 × n + 7.5 bp (38). Meanwhile NRL measurements collectively of different chromatin sources gave rise to the formula ∼10 × n bp (39).

**Figure 9.**
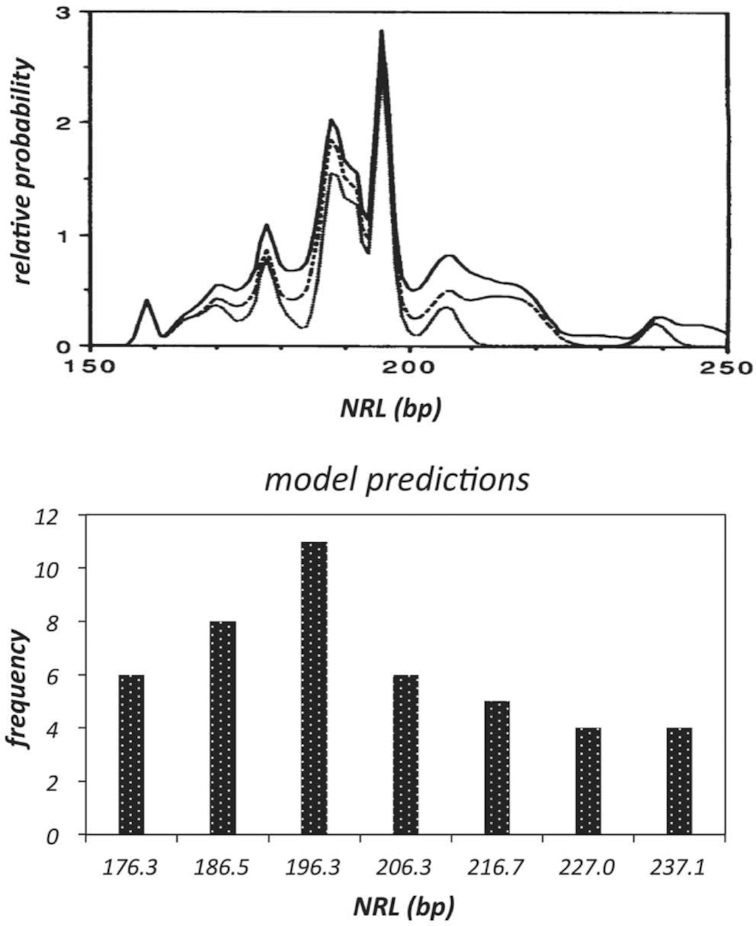
Comparison of linker lengths found *in vivo* and from model predictions. Upper panel: NRL probability distribution derived from measurements of different tissues and organisms (adapted from ref. 39). Lower panel: Occurrence frequency of NRLs (n = 44) in predicted fibres (shown in Figure 8). The labels on x axis are mean values respective of individual bins: 176.3–176.6, 185.9–186.7, 194.8–197.0, 205.0–207.2, 216.0–217.6, 226.8–227.4, 237.0–237.4. Note that the bins are discontinuous. The linker length is modelled continuous although the linker length in terms of bp is an integer.

The rationale for linker DNAs to be at discrete lengths assumes a requirement by higher order chromatin structure that the relative orientations of nucleosomes, affected by linker length, need to be in the correct register with adjacent nucleosomes. For a crossed-linker type model, maintaining the nucleosome orientations with increasing fibre diameter necessitates the linker length to differ by increments of the helical repeat. As expected, predicted fibres (with the twist constraint of the linker DNA imposed) featured high frequencies in ∼147 + 10 × n bp NRLs (n = integer). These results are fully consistent with the NRL probability distribution derived from measurements of different tissues and organisms (39) (Figure 9).

In any case the apparent quantization likely reflects the average values *in vivo*, possible conflicts between linker length and the required nucleosome orientation may be resolved by a slight writhing of linker DNA to alleviate torsional stress. Other influences include the C-terminal domain of linker histone, whose conformation correlates with the configuration of linker DNA (40). Additionally, divalent cations could modulate the intrinsic twist of the linker (see later discussions). Furthermore, recent high resolution crystal structures of nucleosome core particles showed that the length of nucleosomal DNA ranges between 145 and 147 bp (41–43), this finding implies an additional tunability for nucleosome orientations by up to ∼±68.6°.

### Relative orientation of nucleosomes

In the tetranucleosome and reconstituted chromatin fibres the adjacent octamers around their common axis were rotated relative to one another by 22° and 17° respectively (10,12). While a 30° rotation was found between free core particles for optimal stacking involving the H4 N-terminal tail and opposing acidic patch (32). The discrepancies likely result from physical constraints in the chromatin fibre (12–24-mer), and to a lesser extent, in the tetranucleosome (4-mer). Intrinsic to the 2-start structure, the trajectories of the entering and leaving DNAs of each nucleosome having uniform linker lengths are identical, such that the nucleosome symmetry axes relative to entering and leaving DNAs (as in the chromatosome) are close to parallel (close because the entering and leaving DNAs are not in perfect symmetry). If the positions of octamer dyads in this situation coincide with the symmetry axes adjacent octamers would not be in the correct register due to having little relative rotation, but increasing rotation driven by nucleosome stacking would lead to asymmetric distortion of the entering and leaving DNAs, as was seen in the chromatin fibre (12).

**Figure 10.**
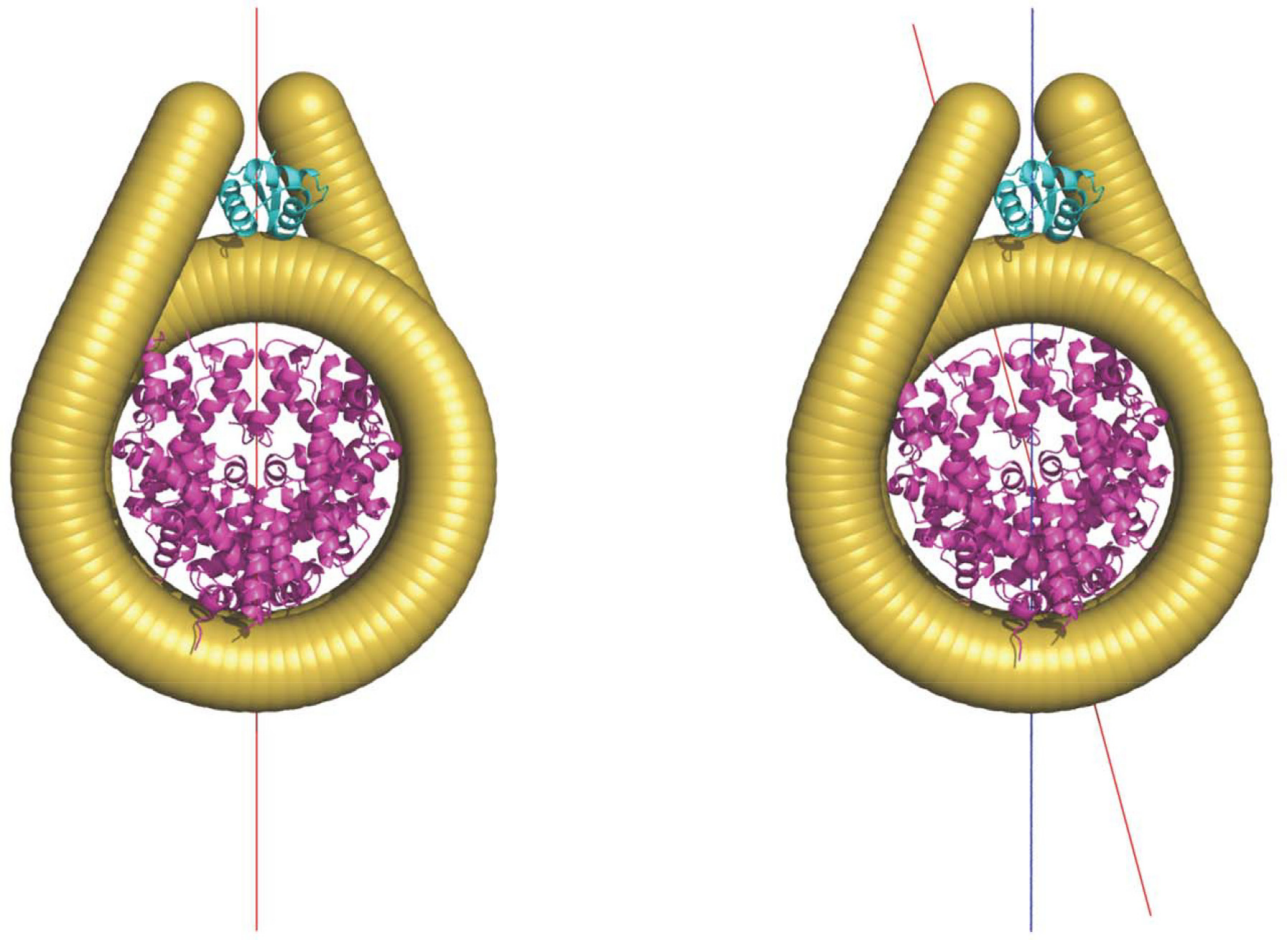
The dyad position relative to entering and leaving DNAs. Left: the position of octamer dyad coincides with the symmetry axis of the nucleosome (determined relative to the entering and leaving DNAs). Right: octamer dyad deviates from the symmetry axis (blue line) of the nucleosome. The red line indicates the dyad axis (determined relative to the octamer). Note that the orientation of linker histone in the diagram was based on the chromatosome structure (PDB ID: 4LQC), where the linker histone resides on the DNA minor groove (45). The binding mode of linker histone will be different (which we have not modelled) where octamer dyad deviates from the symmetry axis (44).

This finding suggests that in a 2-start structure with optimal nucleosome stacking, the relative rotation of adjacent octamers should readily occur while the symmetry axes relative to entering and leaving DNAs remain parallel. In other words, the octamer dyad does not coincide with the symmetry axis (Figure 10). In contrast, the opposite is true in the 1-start model (4) because the relative rotation between stacked nucleosomes is intrinsic to the model such that the adjacent octamers are readily in the correct register for stacking. It follows that the linker histone assumes on-dyad binding in a 1-start configuration (Figure 10). In a 187 bp NRL short 2-start oligonucleosomal arrays assuming a 2-start structure both crystallography and hydroxyl-radical footprinting indicate an on-dyad linker histone orientation (13,44). However, the nucleosomes form parallel stacks in the crystal whereas in cryo-EM structures having twisted stacks an off-dyad linker histone is apparent (12,13). Nonetheless, in isolated chromatosomes NMR studies indicates that *Drosophila* linker histone H1 and chicken H5 have, respectively, preferred off- and on-dyad locations (45–48). These linker histones are associated *in vivo*, respectively, with shorter (49) and longer NRLs. Variation in linker histone dyad location would also likely impact on the configuration of the chromatosome stem structure (50,51).

### Topology of fibre DNA

**Figure 11.**
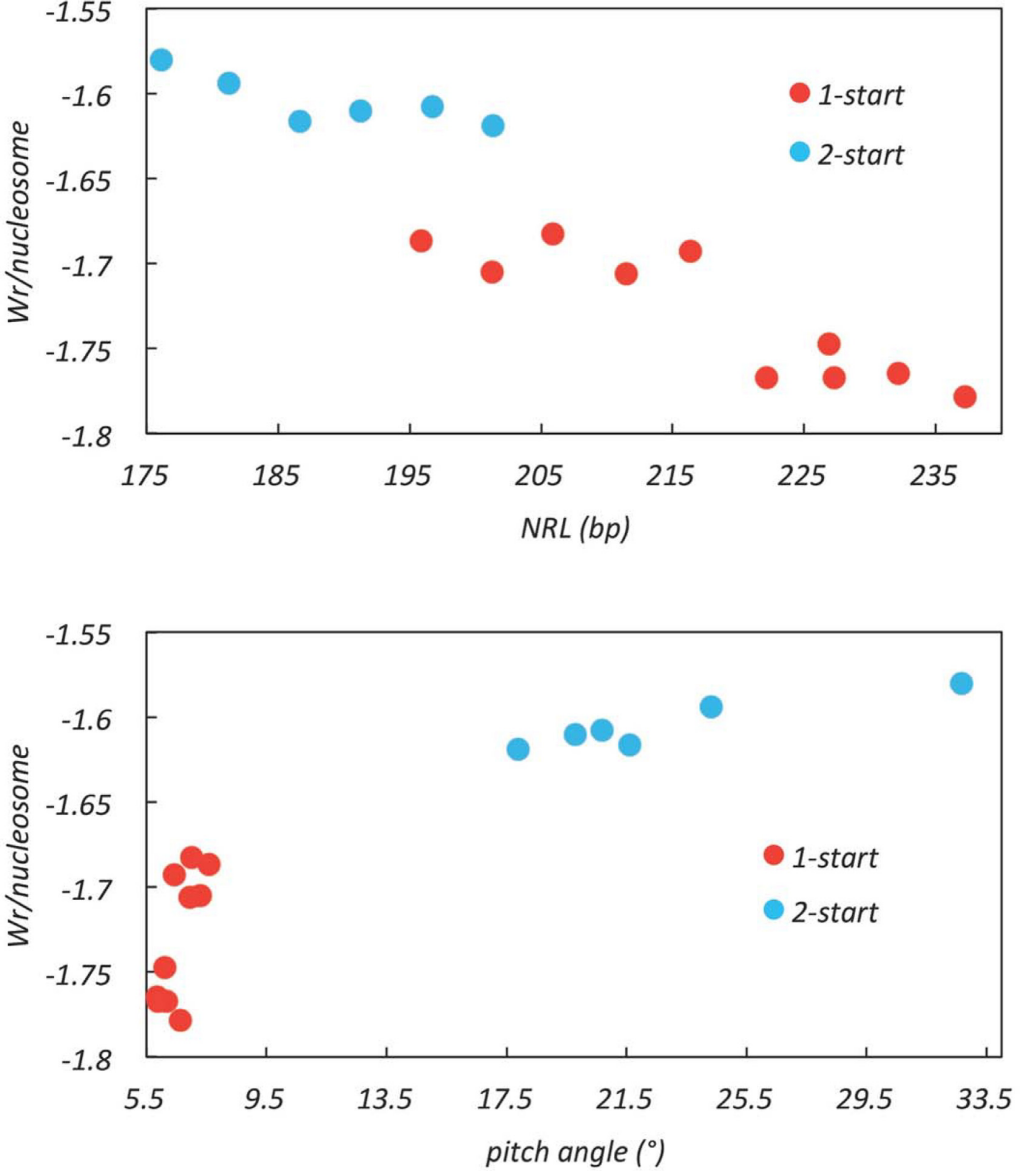
The DNA writhe packaged in compact fibres. The total writhe of fibre DNA was calculated for compact fibres using the 32-mer structures. The writhe per nucleosome was plotted against the pitch angle of nucleosome stack and nucleosome repeat length (NRL) respectively. Data points corresponding to 2-start and 1-start models were indicated in blue and red respectively. The data points include fibres with uniform NRLs and those with mixed NRLs (Table 1).

Our modelling methodology describes the path of the DNA in the fibres with respect to the positions of individual base-pairs, thus approximating to a smooth space-curve. Using these parameters we computed the total DNA writhe for the fibres with quantised NRLs between ∼177 and ∼237 bp. Fibres with shorter NRLs (167 bp) have a lower stoichiometry of linker histone binding (20) and so were omitted. We find, first, that for fibres the constrained writhe becomes more negative with increasing NRL and that this increase correlates with a decreasing pitch angle for the nucleosome stacks (Figure 11). The value of the writhe/nucleosome increases from *−*1.58 for the 177 bp NRL 2-start fibre to *−*1.78 for the 237 bp NRL 1-start fibre. Notably, the values of the writhe associated with the 1- and 2-start 197 bp NRL fibres, *−*1.69 and *−*1.61 respectively correspond to changes in ΔL of *−*1.37 and *−*1.45, values very similar to the experimentally observed total change in linking number, *−*1.4, required for the maximal chromatin compaction of a 38 × 200 bp nucleosome array achieved with magnetic tweezers (24). These numbers imply that for a regular nucleosome array the average constrained writhe/nucleosome will be less negative for shorter NRLs and would be consistent with an active role of linker DNA in fibre compaction.

Additionally, we find that for the same NRL the 1-start configuration constrains more negative writhe than the 2-start fibres. This difference correlates with an altered nucleosome connectivity (stacking neighbours respective to their relative positions in the extended array) of the 1-start configuration and with a consequent closer approach of the entering and exiting linker DNAs in the 1-start fibre relative to the 2-start fibre. We conclude that the formation of a 1-start fibre is consistent with experimental topological determinations of fibre folding.

### The topological contribution of linker DNA

In a 197 bp NRL compact fibre our calculated value of ΔWr/nucleosome is very similar to the average experimental value of ΔL determined for the constraint of a single chromatosome on a small DNA circle (52). The latter authors suggested that any contribution of changes in intrinsic twist to the DNA topology of compact fibres is small or negligible. Since ΔL, the change in linking number, is the sum of both ΔWr and ΔTw we cannot necessarily assume that the twist of linker DNA is unchanged simply by extrapolation from a small DNA circle to a fibre. However for a particular extent of compaction the value of ΔL/fibre is independent of the precise path of the linker DNA (53) and thus also of the partition between twist and writhe. Assuming that the intrinsic twist, and hence the writhe, of nucleosomal DNA varies little during fibre compaction we can ask whether changes in the twist of the linker DNA between consecutive chromatosomes are consistent with our model.

**Figure 12.**
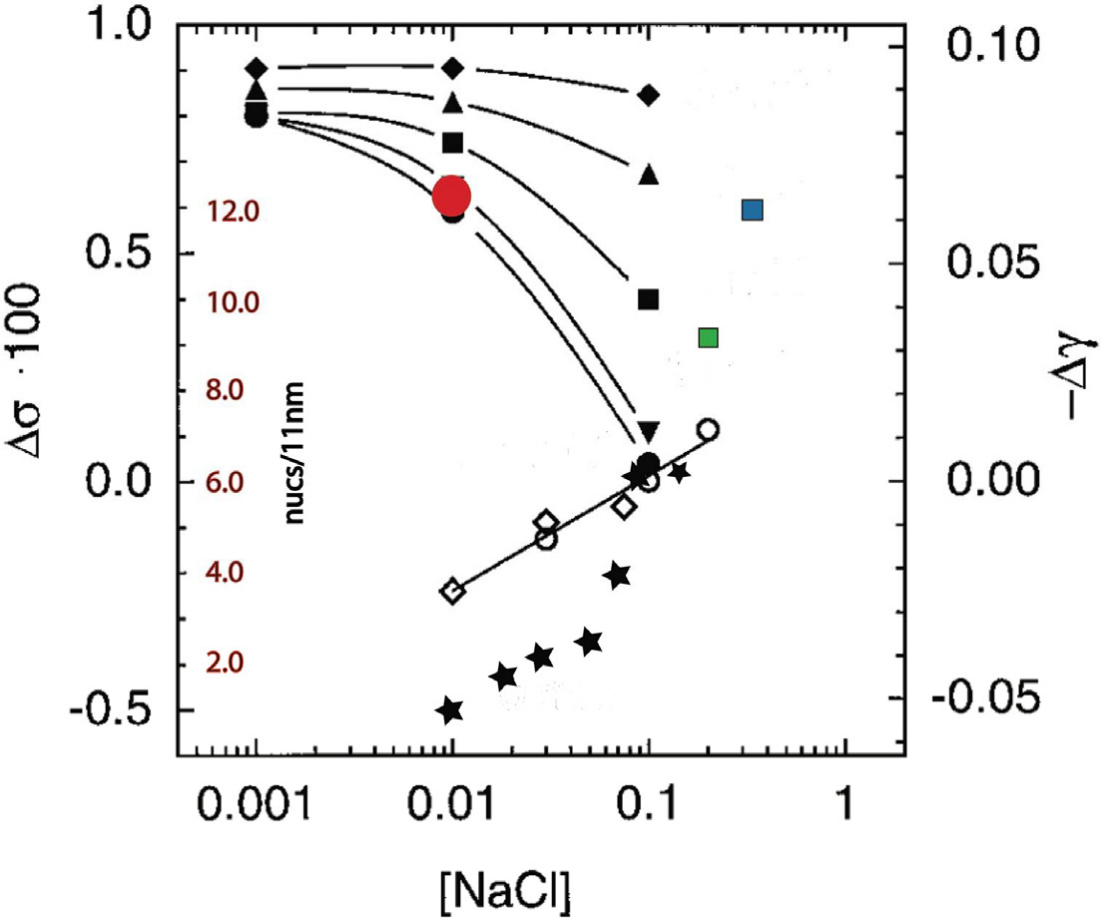
Correlation of cation-dependent change in DNA helical repeat adapted from (28) and fibre density (14,15,19,26). These data are plotted according to the X^+^ and Mg^2+^ concentrations used. Where appropriate (i.e. when K^+^ was used instead of Na^+^) X^+^ is plotted in terms of Na^+^ equivalents according to ref. 27). Symbols from ref. 28): open diamonds, filled circles, inverted triangles, squares, triangles, filled diamonds - [Na+] with successively 0, 0.5, 1, 2, 5 mM [Mg^2+^]; fibre data: filled stars, red circle, green square, blue square from, respectively, (26,19,14,15). Note that the data from (26) indicate a 2-phase folding pathway as further described in Supplementary Figure S3.

Three types of experimental observation suggest that the intrinsic twist of linker DNA may change during fibre compaction. First, the ability of the C-terminal H1 tail to condense DNA (30,54–56) would, if replicated in the fibre, alter the twist constraints on linker DNA imposed by an aqueous environment. Second, both cation-induced DNA condensation and dinucleosome compaction are reversed by ethidium bromide, a DNA intercalator which untwists DNA (57,58). Third, the salt-dependent folding of the 30-nm fibre is strongly dependent on both the concentration and nature of solution cations (8,25). Increasing ionic strength not only decreases charge repulsion between DNA duplexes in close proximity but also alters the intrinsic helical repeat of DNA in a cation-dependent manner (27–29). In particular, on a molar basis Mg^2+^, which favours the formation of more compact fibres (8,19) decreases the helical repeat 3–4× more effectively than Na^+^ (27–29). Combining the data of Rybenkov *et al.* (28) with previous experimental determinations of fibre density using linker histone H5, we find a strong correspondence between the cation-induced decrease in helical repeat and fibre folding (Figure 12). Taken together these observations imply that, in a fibre, linker DNA is likely overtwisted (57). This has two consequences. First, an increased twist of linker DNA induced by the H1 C-terminal tail in concert with external cations would act as an internal torsional driver of fibre coiling and compaction, analogous to the experimental situation of external magnetic tweezers. Second the helical repeat of the linker DNA would likely define the increment in preferred NRLs. For compact fibres our modelling implies that the quantised linker lengths are highly preferred. Following Yao *et al.* (57) we suggest that, in a manner analogous to chromatin remodelers, applied internal torsion can enable limited reshuffling of nucleosome positions to create a more regular array, always providing that any energetic barriers imposed by strong positioning can be overcome. Accordingly, less compact fibres would, as observed, be more structurally heterogeneous (5,26,59,60).

The coiling of the fibre in response to overtwisting of linker DNA would be a direct Tw to Wr compensation (53). To examine whether this is compatible with our model we have tested whether, in a closed topological system, decreasing the helical repeat of the linker DNA is reflected in corresponding changes in ΔWr. We find that this condition is met (Figure S1 and movie S3 in Supplementary Data). We also note that the total torsion resulting from linker overtwisting will be proportional to linker length, assuming an equivalent charge neutralisation.

The change in DNA topology accompanying the folding of an extended nucleosome array to a fully compact fibre can be described by the formal relationship:

$$\begin{equation*}\Delta {\rm{L}} = \Delta {\rm{Tw}} + \Delta {\rm{Wr}}\end{equation*}$$

where L = linking number, Tw = intrinsic twist and Wr = writhe (61)

If we then assume that the additional writhe of the fibre, i.e. excluding the writhe attributable to individual nucleosomes, derives from topological compensation we infer that the helical repeat of linker DNA in compact fibres to be ∼10 bp/turn (Supplementary Figure S2). This corresponds to a value of ΔL of approximately *−*1.4/nuc (Table 1), in good agreement with previous theoretical (33,53) and experimental studies (24).

Is our modelling consistent with the notion that a 2-start to 1-start transition could facilitate a phase transition in compact 30-nm fibres where the linker DNA adopts a condensed state analogous to that in cation-induced toroidal structures? Our model invokes a short centre-to-centre distance for adjacent linker DNAs (4) corresponding to an approximate doubling of the DNA concentration of linker DNA on intercalation to ∼400–500 mg/ml in the interior of the fibre (62). Experimental determinations of this distance in protein-free condensed DNA and nucleoprotamine filaments reveal minimum separations in the range of 1.8–2.7 nm (63–66). A linker helical repeat of ∼10 bp/turn would facilitate the alignment of closely packed duplexes (67). Since by itself the C-terminal tail of linker histone H1 is known to induce DNA condensation with long range order (30) we propose that the 2-start to 1-start intercalation could further compact linker DNA by increasing the local linker DNA concentration and thereby promoting a structural transition between condensed DNA mesophases (66,68). On this model the condensation of linker DNA during the folding of a long NRL chromatin fibre proceeds from an observed initial weak long-range cholesteric order (69) to a more compact form - possibly line-hexagonal (66) - in isolated fibres from avian erythrocytes and echinoderm sperm chromatin. Such a transition could contribute to the stability of the 1-start configuration. The topology we have described is consistent with a toroidal geometry for the 30-nm fibre but by itself does not address the question of the higher-order organisation of these fibres (70).

### *In vivo* relevance

We have argued that the structure and topology assumed by the 30-nm fibre are intimately related and dependent on two principal parameters related to linker DNA - its local concentration in the interior of the fibre and its intrinsic helical repeat. More generally, these parameters have implications for chromatin structure *in vivo*. We have modelled the structure of a fibre which contains only histones whereas *in vivo* the fibre is associated with other abundant chromatin associated proteins - for example, HMG proteins. While histone-bound DNA is relaxed or more often slightly overtwisted, HMG-B proteins untwist DNA (31). Binding of HMG-B proteins to linker DNA would thus increase its helical repeat, in effect favouring a looser structure than the coiled 30-nm fibre. To this extent we feel that our model is consistent with the *in vivo* observations reviewed by Maeshima *et al.* (71). Furthermore the most compact fibres, in terms of linker DNA concentration/rise, observed *in vivo* are those found in terminally differentiated echinoderm sperm (14,16). These fibres also have the longest NRLs which, in our calculations, correlate with the lowest (most twisted) helical repeat of linker DNA. Analogously compact fibre formation *in vitro* used linker histone H5 from terminally differentiated avian erythrocytes (19).

Our model also implies that the overtwisting of the linker DNA and hence the structure of the fibre can potentially be fine-tuned by epigenetic modifications of the C-terminal tail. One example, of many (reviewed in (72)), would be the CDK-mediated phosphorylation of SPKK sequences during mitosis. In a model *in vitro* system this phosphorylation results in the loss of long range chiral order in a condensed C-terminal tail-DNA complex (30) while still retaining the ability to form coacervates (30,56). *In vivo* such an effect would imply that metaphase chromosomes could, as observed, lack 30-nm fibres (73,74). Conversely, maturation of both echinoderm sperm (75) and avian erythrocytes (76) is accompanied by H1 C-terminal tail dephosphorylation. The topology of the fibre, and hence the ability to form a 30-nm structure, would also be directly dependent on the relative exiting and entry angles of the linker DNA. The histone variant CENP-A, associated with centromeres, has a diminished tendency to stabilise negative crossovers in linker DNA (77,78). Similarly nucleosomes containing the variant H2A-B (aka H2A-Bbd) lacking a linker histone docking domain (79–81) would for similar reasons likely preclude 30-nm fibre formation, although in this case in a fibre with a more open structure.

### Relation to previous work

#### Fibre models

The path of the linker DNA in chromatin fibres has traditionally been described by the ‘two-angle model’ (82). This model describes the chromatin fibre geometry in terms of the entry-exit angle of the nucleosomal DNA and the rotational setting of the opposing nucleosomes with respect to each other. Crucially the linker DNA remains straight. Our model calculates fibre topology independently of the assumptions of the two-angle model but we note that by altering the twist of the linker DNA any writhing of the linker would likely be minimized.

The lack of a precise experimental description of the trajectory of linker DNA in chromatin fibres has restricted attempts to model 30-nm fibre structure and to calculate fibre DNA topology. The original helical 1-start model for compact fibres proposed by Finch and Klug (5) was predicated on the observation of a low fibre pitch angle. Since then many different fibre structures have been proposed but only one, the crossed-linker 2-start model has received direct experimental confirmation from crystal and cryo-EM studies and then only for fibres with densities of ≤6–7 nucleosomes/11 nm (10,12,13). Although Finch and Klug (5) visualized a mixture of both low and high pitch fibres consistent respectively with 1-start and 2-start helices, they argued that the latter were artefactual.

For fibres of similar diameter the pitch of the coiled nucleosome stacks is directly related to helix order (1-start, 2-start, multi-start, etc), provided that the structure of the core nucleosome is invariant. For compact fibres three 1-start models have been proposed: the solenoid (5), and two variants of the intercalation of the two stacks of a 2-start structure to form a single coil (4,9). Both the latter are compatible with a mixed population of 1- and 2-start fibres but the intercalation of a 2-start crossed-linker structure (4) permits greater compaction than the intercalation of a 2-start twisted ribbon structure (9). A significant limitation of the solenoid model is that any transition between 1-start and 2–start fibres with the same NRL is obliged to proceed via an unfolded nucleosome array. High compaction can also be achieved in multi-start models (19,83–86) but in these cases the intrinsic high pitch of the nucleosome stacks is inconsistent with Finch and Klug's original observation of low pitch fibres.

A novel variant of 2-start models is the ‘gaping’ proposal of Mozziconacci and Victor (32). In this model compaction is accompanied by an untwisting of nucleosomal DNA, ‘gaping’, resulting in a reduction in fibre pitch while retaining a 2-start crossed-linker helical organisation. Although the ‘gaping’ transition is topologically equivalent to ours, experimental evidence suggests that it is more likely to occur under low-salt conditions which would disfavour fibre compaction (87).

#### Fibre topology

Zhurkin and collaborators (33,88) have recently modelled low density 2-start fibres and calculated that the most compact 2-start fibres for NRLs in the 10n series have an NRL of ∼185 bp. This observation would be consistent with our model proposing that the additional compaction of fibres with longer NRLs is potentially associated with a structural transition to a 1-start structure.

Our fibre model predicts high fibre densities (≥10 nucs/11 nm) consistent with those experimentally observed by Bartolomé *et al.* (89) and by Robinson *et al.* (19) but is inconsistent with an apparent limit density of ∼ 6–7 nucs/11 nm (26,59). As originally highlighted by Widom (8) this apparent experimental discrepancy is likely a consequence of the nature of the cation(s) used to drive compaction. Whereas in the presence of most concentrations of Na^+^ alone only fibres of 6–7 nucs/11 nm are observed, the presence of Mg^2+^ and the absence of significant monovalent cation concentrations favours the formation of higher density fibres (8). These results mirror the effects of these cations on changes in the superhelical parameters of free DNA mediated by alteration of its intrinsic twist (27–29) (see also Supplemetary Data).

Finally, to what extent are our conclusions compatible with other experimental measurements of the topology of compact chromatin? In 1978 Müller *et al.* (90) reported that removal of linker histone from ‘compact’ chromatin formed on circular SV40 DNA resulted in no change in linking number. These experiments measured the sum of both constrained (by histones) and unconstrained (in free DNA) superhelicity in a closed topological system and consequently removal of H1 would not, by itself, change the linking number. In a separate experiment, using nicking-closing enzyme, the authors showed that the constrained supercoils in the absence of H1, corresponded to the average number of core particles formed on SV40 DNA (see also ref. 91 ). In the context of our conclusions we surmise that the SV40 DNA with compact chromatin the topology of SV40 DNA has two components - negative superhelicity constrained by both the core histones and histone H1 and also unconstrained positive superhelicity that compensates for any linker histone dependent negative superhelicity.

A similar notion underlies the release of negative superhelicity observed when compact chromatin unfolds *in vivo* (92). Here the initial H1-induced compaction would be accompanied by the formation of unconstrained positive supercoils beyond the fibre limits. Such positive superhelicity would be relaxed by topoisomerase II *in vivo* thereby allowing the fibre to release negative superhelicity on decompaction.

## CONCLUSION

In 1976, Finch and Klug reported compact chromatin fibres with a low helical pitch. On this basis they proposed that nucleofilaments condensed to form a ‘solenoid’ in which spatially adjacent nucleosomes stacked in a 1-start helix, arguing that fibres with 2-start stacking would have a higher pitch angle. Since then overwhelming experimental evidence has demonstrated the existence of 2-start, but less compact, chromatin fibres (10–13). Recently we proposed that compact 1-start fibres could be formed by the intercalation of the nucleosome stacks within less compact 2-start fibres with longer NRLs while retaining the trajectory of the linker DNA across the interior of the fibre (4). In this paper, we have devised a simple method to test whether the modelled structures of compact crossed-linker 1-start fibres are consistent with reported experimental parameters. We find that the orientation of octamer dyad altered by intercalation in 1-start fibres likely requires a different binding mode of the globular domain of the linker histone similar to that adopted by histone H5 and human H1.5 (44,46). It is also accompanied by a step increase in constrained DNA writhe related to a concomitant sharp reduction in the helical pitch of the chromatin fibre. Another important consequence of the intercalation is an increase in the local concentration of linker DNA which we propose could facilitate a phase transition to more condensed DNA packing. We also show that the salt dependent coiling of the fibre is likely accompanied by a decrease in the helical repeat of linker DNA, mediated by electrolyte cations and the C-terminal tail of the linker histone. This increased (right-handed) twist of the linker DNA could then contribute in large part to driving the left-handed coiling (writhe) of the fibre. We conclude that the modelled structure of a 1-start crossed-linker chromatin fibre is consistent with the reported physical properties of the most compact fibres and provides a biological rationale for DNA protection in the isolated chromatin fibres from the transcriptionally inactive avian erythrocytes and echinoderm sperm.

## Supplementary Material

gkz495_Supplemental_Files

## References

[1] Ausió J. The shades of gray of the chromatin fiber. Bioessays. 2015; 37:46–51. 25328133 10.1002/bies.201400144

[2] Zhu P., Li G Higher-order structure of the 30-nm chromatin fiber revealed by cryo-EM. IUBMB Life. 2016; 68:873–878. 27704715 10.1002/iub.1568

[3] Williams S.P., Athey B.D., Muglia L.J., Schappe R.S., Gough A.H., Langmore J.P. Chromatin fibres are left-handed double helices with diameter and mass per unit length that depend on linker length. Biophys. J. 1986; 49:233–248. 3955173 10.1016/S0006-3495(86)83637-2 PMC1329627

[4] Wu C., McGeehan J.E., Travers A. A metastable structure for the compact 30 nm fibre. FEBS Lett. 2016; 590:935–942. 26969895 10.1002/1873-3468.12128 PMC4863496

[5] Finch J.T., Klug A. A solenoidal model for superstructure in chromatin. Proc. Natl. Acad. Sci. U.S.A. 1976; 73:1897–1901. 1064861 10.1073/pnas.73.6.1897 PMC430414

[6] Widom J., Klug A. Structure of the 300 Å chromatin filament: X-ray diffraction from oriented samples. Cell. 1985; 43:207–213. 4075395 10.1016/0092-8674(85)90025-x

[7] Widom J., Finch J.T., Thomas J.O. Higher-order structure of long repeat chromatin. EMBO J. 1985; 4:3189–3194. 4092681 10.1002/j.1460-2075.1985.tb04064.x PMC554641

[8] Widom J. Physicochemical studies of the folding of the 100 A nucleosome filament into the 300 A filament: cation dependence. J. Mol. Biol. 1986; 190:411–424. 3783706 10.1016/0022-2836(86)90012-4

[9] Sen D., Mitra S, Crothers D.M. Higher order structure of chromatin: evidence from photochemically detected linear dichroism. Biochemistry. 1986; 25:3441–3447. 3730368 10.1021/bi00359a052

[10] Schalch T., Duda S., Sargent D.F., Richmond T.J. X-ray structure of a tetranucleosome and its implications for the chromatin fibre. Nature. 2005; 436:138–141. 16001076 10.1038/nature03686

[11] Scheffer M.P., Eltsov M., Frangakis A.S. Evidence for short-range helical order in the 30-nm chromatin fibres of erythrocyte nuclei. Proc. Natl. Acad. Sci. U.S.A. 2011; 108:16992–16997. 21969536 10.1073/pnas.1108268108 PMC3193215

[12] Song F., Chen P., Sun D., Wang M., Dong L., Liang D., Xu R.M., Zhu P., Li G. Cryo-EM study of the chromatin fibre reveals a double helix twisted by tetranucleosomal units. Science. 2014; 344:376–380. 24763583 10.1126/science.1251413

[13] Garcia-Saez I., Menoni H., Boopathi R., Shukla M.S., Soueidan L., Noirclerc-Savoye M., Le Roy A., Skoufias D.A., Bednar J., Hamiche A. et al. Structure of an H1-bound 6-nucleosome array reveals an untwisted two-start chromatin fiber conformation. Mol. Cell. 2018; 72:902–915. 30392928 10.1016/j.molcel.2018.09.027

[14] Woodcock C.L., Frado L.L., Rattner J.B. The higher-order structure of chromatin: evidence for a helical ribbon arrangement. J. Cell Biol. 1984; 99:42–52. 6736132 10.1083/jcb.99.1.42 PMC2275637

[15] Williams S.P., Langmore J.P. Small angle x-ray scattering of chromatin. Radius and mass per unit length depend on linker length. Biophys. J. 1991; 59:606–618. 2049522 10.1016/S0006-3495(91)82276-7 PMC1281225

[16] Giannasca P.J., Horowitz R.A., Woodcock C.L. Transitions between in situ and isolated chromatin. J. Cell Sci. 1993; 105:551–561. 8408284 10.1242/jcs.105.2.551

[17] Scheffer M.P., Eltsov M., Bednar J., Frangakis A.S. Nucleosomes with aligned dyads are found in native chromatin in vivo. J. Struct. Biol. 2012; 178:207–214. 22138167 10.1016/j.jsb.2011.11.020

[18] Gilbert N., Allan J. Distinctive higher-order chromatin structure at mammalian centromeres. Proc. Natl. Acad. Sci. U.S.A. 2001; 98:11949–11954. 11593003 10.1073/pnas.211322798 PMC59820

[19] Robinson P.J.J., Fairall L., Huynh V.A.T., Rhodes D. EM measurements define the dimensions of the “30-nm” chromatin fibre: evidence for a compact, interdigitated structure. Proc. Natl. Acad. Sci. U.S.A. 2006; 103:6506–6511. 16617109 10.1073/pnas.0601212103 PMC1436021

[20] Routh A., Sandin S., Rhodes D Nucleosome repeat length and linker histone stoichiometry determine chromatin fiber structure. Proc. Natl. Acad. Sci. U.S.A. 2008; 105:8872–8877. 18583476 10.1073/pnas.0802336105 PMC2440727

[21] Levitt M. Protein folding by restrained energy minimization and molecular dynamics. J. Mol. Biol. 1983; 170:723–764. 6195346 10.1016/s0022-2836(83)80129-6

[22] Klenin K., Langowski J. Computation of writhe in modeling of supercoiled DNA. Biopolymers. 2000; 54:307–317. 10935971 10.1002/1097-0282(20001015)54:5<307::AID-BIP20>3.0.CO;2-Y

[23] Agarwal P.K., Edelsbrunner H., Wang Y. Computing the writhing number of a polygonal knot. Proc. 13th ACM-SIAM Sympos. 2002; Discrete Alg. (SoDA) 791–799.

[24] Recouvreux P., Lavelle C., Barbi M., Condé e Silva N., Le Cam E., Victor J.M., Viovy J.L Linker histones incorporation maintains chromatin fiber plasticity. Biophys. J. 2011; 100:2726–2735. 21641318 10.1016/j.bpj.2011.03.064 PMC3117191

[25] Thoma F., Koller T., Klug A. Involvement of histone H1 in the organization of the nucleosome and of the salt-dependent superstructures of chromatin. J. Cell Biol. 1979; 83:403–427. 387806 10.1083/jcb.83.2.403 PMC2111545

[26] Gerchman S.E., Ramakrishnan V. Chromatin higher-order structure studied by neutron scattering and scanning transmission electron microscopy. Proc. Natl. Acad. Sci. U.S.A. 1987; 84:7802–7806. 3479765 10.1073/pnas.84.22.7802 PMC299397

[27] Anderson P., Bauer W. Supercoiling in closed circular DNA: dependence on ion type and concentration. Biochemistry. 1978; 17:594–601. 623732 10.1021/bi00597a006

[28] Rybenkov V.V., Vologodskii A.V., Cozzarelli N.R. The effect of ionic conditions on DNA helical repeat, effective diameter and free energy of supercoiling. Nucleic Acids Res. 1997; 25:1412–1418. 9060437 10.1093/nar/25.7.1412 PMC146597

[29] Xu Y-C., Bremer H. Winding of the DNA helix by divalent metal ions. Nucleic Acids Res. 1997; 25:4067–4071. 9321659 10.1093/nar/25.20.4067 PMC147022

[30] Turner A.L., Watson M., Wilkins O.G., Cato L., Travers A., Thomas J.O., Stott K. Highly disordered histone H1-DNA model complexes and their condensates. Proc. Natl. Acad. Sci. U.S.A. 2018; 115:11964–11969. 30301810 10.1073/pnas.1805943115 PMC6255207

[31] Murphy F.V 4th, Sweet R.M., Churchill M.E.A. The structure of a chromosomal high mobility group protein-DNA complex reveals sequence-neutral mechanisms important for non-sequence-specific DNA recognition. EMBO J. 1999; 18:6610–6618. 10581235 10.1093/emboj/18.23.6610 PMC1171724

[32] Mozziconacci J., Victor J.M. Nucleosome gaping supports a functional structure for the 30 nm chromatin fibre. J. Struct. Biol. 2003; 143:72–76. 12892727 10.1016/s1047-8477(03)00102-3

[33] Norouzi D., Zhurkin V.B. Topological polymorphism of the two-start chromatin fibre. Biophys. J. 2015; 108:2591–2600. 25992737 10.1016/j.bpj.2015.04.015 PMC4457244

[34] Lohr D., van Holde K.E. Organization of spacer DNA in chromatin. Proc. Natl. Acad. Sci. U.S.A. 1979; 76:6326–6330. 392519 10.1073/pnas.76.12.6326 PMC411857

[35] Wang J.P., Fondufe-Mittendorf Y., Xi L., Tsai G.F., Segal E., Widom J. Preferentially quantised linker DNA lengths in Saccharomyces cerevisiae. PLoS Comp. Biol. 2008; 4:1–10. 10.1371/journal.pcbi.1000175 PMC2522279 18787693

[36] Cui F., Zhurkin V.B. Distinctive sequence patterns in metazoan and yeast nucleosomes: implications for linker histone binding to AT-rich and methylated DNA. Nucleic Acids Res. 2009; 37:2818–2829. 19282449 10.1093/nar/gkp113 PMC2685081

[37] Strauss F., Prunell A. Organization of internucleosomal DNA in rat liver chromatin. EMBO J. 1983; 2:51–56. 11894908 10.1002/j.1460-2075.1983.tb01379.x PMC555085

[38] Kato M., Onishi Y., Wada-Kiyama Y., Abe T., Ikemura T., Koqan S., Bolshoy A., Trifonov E.N., Kiyama R. Dinucleosome DNA of human k562 cells: experimental and computational characterizations. J. Mol. Biol. 2003; 332:111–125. 12946351 10.1016/s0022-2836(03)00838-6

[39] Widom J. A relationship between the helical twist of DNA and the ordered positioning of nucleosomes in all eukaryotic cells. Proc. Natl. Acad. Sci. U.S.A. 1992; 89:1095–1099. 1736292 10.1073/pnas.89.3.1095 PMC48392

[40] Fang H., Wei S., Lee T.H., Hayes J.J. Chromatin structure-dependent conformations of the H1 CTD. Nucleic Acids Res. 2016; 44:9131–9141. 27365050 10.1093/nar/gkw586 PMC5100576

[41] Davey C.A., Sargent D.F., Luger K., Maeder A.W., Richmond T.J. Solvent mediated interactions in the structure of the nucleosome core particle at 1.9 Å resolution. J. Mol. Biol. 2002; 319:1097–1113. 12079350 10.1016/S0022-2836(02)00386-8

[42] Richmond T.J., Davey C.A. The structure of DNA in the nucleosome core. Nature. 2003; 423:145–150. 12736678 10.1038/nature01595

[43] Vasudevan D., Chua E.Y., Davey C.A. Crystal structures of nucleosome core particles containing the ‘601’ strong positioning sequence. J. Mol. Biol. 2010; 403:1–10. 20800598 10.1016/j.jmb.2010.08.039

[44] Syed S.H., Goutte-Gattat D., Becker N., Meyer S., Shukla M.S., Hayes J.J., Everaers R., Angelov D., Bednar J., Dimitrov S. Single-base resolution mapping of H1-nucleosome interactions and 3D organization of the nucleosome. Proc. Natl. Acad. Sci. U.S.A. 2010; 107:9620–9625. 20457934 10.1073/pnas.1000309107 PMC2906896

[45] Zhou B.R., Feng H., Kato H., Dai L., Yang Y., Zhou Y., Bai Y. Structural insights into the histone H1-nucleosome complex. Proc. Natl. Acad. Sci. U.S.A. 2013; 110:19390–19395. 24218562 10.1073/pnas.1314905110 PMC3845106

[46] Zhou B.R., Jiang J., Feng H., Ghirlando R., Xiao T.S., Bai Y. Structural mechanisms of nucleosome recognition by linker histones. Mol. Cell. 2015; 59:1–11. 26212454 10.1016/j.molcel.2015.06.025 PMC4546531

[47] Zhou B.R., Feng H., Ghirlando R., Li S., Schwieters C.D., Bai Y. A small number of residues can determine if linker histones are bound on or off dyad in the chromatosome. J. Mol. Biol. 2016; 428:3948–3959. 27558112 10.1016/j.jmb.2016.08.016 PMC6291011

[48] Öztürk M.A., Cojocaru V., Wade R.C. Dependence of chromatosome structure on linker histone sequence and posttranslational modification. Biophys. J. 2018; 114:2363–2375. 29759374 10.1016/j.bpj.2018.04.034 PMC6129471

[49] Baldi S., Krebs S., Blum H., Becker P.B. Genome-wide measurement of local nucleosome array regularity by nanopore sequencing. Nat. Struct. Mol. Biol. 2018; 25:894–901. 30127356 10.1038/s41594-018-0110-0

[50] Hamiche A., Schultz P., Ramakrishnan V., Oudet P., Prunell A Linker histone-dependent DNA structure in linear mononucleosomes. J. Mol. Biol. 1996; 257:30–42. 8632457 10.1006/jmbi.1996.0144

[51] Meyer S., Becker N.B., Syed S.H., Goutte-Gattat D., Shukla M.S., Hayes J.J., Angelov D., Bednar J., Dimitrov S., Everaers R. From crystal and NMR structures, footprints and cryo-electron micrographs to large and soft structures: nanoscale modelling of the nucleosomal stem. Nucleic Acids Res. 2011; 39:9139–9154. 21835779 10.1093/nar/gkr573 PMC3241633

[52] Zivanovic Y., Duband-Goulet I., Schultz P., Stofer E., Oudet P., Prunell A. Chromatin reconstitution on small DNA rings. III. Histone H5 dependence of DNA supercoiling in the nucleosome. J. Mol. Biol. 1990; 214:479–495. 2166168 10.1016/0022-2836(90)90195-R

[53] Barbi M., Mozziconacci J., Wong H., Victor J.M. DNA topology in chromosomes: a quantitative survey and it physiological implications. J. Math. Biol. 2014; 68:145–179. 23179130 10.1007/s00285-012-0621-y

[54] Clark D.J., Thomas J.O. Salt-dependent co-operative interactions of histone H1 and DNA. J. Mol. Biol. 1986; 187:569–580. 3712436 10.1016/0022-2836(86)90335-9

[55] Clark D.J., Thomas J.O. Differences in the binding of H1 variants to DNA. Cooperativity and linker-length related distribution. Eur. J. Biochem. 1988; 178:225–233. 3203690 10.1111/j.1432-1033.1988.tb14447.x

[56] Roque.A, Ponte I., Arrondo J.L., Suau P. Phosphorylation of the carboxy-terminal domain of histone H1: effects on secondary structure and DNA condensation. Nucleic Acids Res. 2008; 36:4719–2476. 18632762 10.1093/nar/gkn440 PMC2504289

[57] Yao J., Lowary P.T., Widom J. Twist constraints on linker DNA in the 30-nm chromatin fiber: implications for nucleosome phasing. Proc. Natl. Acad. Sci. U.S.A. 1993; 90:9364–9368. 8415708 10.1073/pnas.90.20.9364 PMC47568

[58] Widom J, Baldwin R.L. Inhibition of cation-induced DNA condensation by intercalating dyes. Biopolymers. 1983; 22:1621–1632. 6223671 10.1002/bip.360220613

[59] Ghirlando R., Felsenfeld G. Hydrodynamic studies on defined heterochromatin fragments support a 30-nm fiber having six nucleosomes per turn. J. Mol. Biol. 2008; 376:1417–1425. 18234217 10.1016/j.jmb.2007.12.051 PMC2774144

[60] Correll S.J., Schubert M.H., Grigoryev S.A. Short nucleosome repeats impose rotational modulations on chromatin fibre folding. EMBO J. 2012; 31:2416–2426. 22473209 10.1038/emboj.2012.80 PMC3364735

[61] White J.H. Self-Linking and the Gauss integral in higher dimensions. Am. J. Math. 1969; 91:693–728.

[62] Podgornik R., Strey H.H., Gawrisch K., Rau D.C., Ruprect A., Parsegian V.A. Bond orientational order, molecular motion, and free energy of high-density DNA mesophases. Proc. Natl. Acad. Sci. U.S.A. 1996; 93:4461–4266. 10.1073/pnas.93.9.4261 PMC39523 8633052

[63] Böttcher C., Endish C., Fuhrhop J.H., Caterall C., Eaton M. High-yield preparation of oligomeric C-type DNA toroids and their characterisation by cryoelectron microscopy. J. Am. Chem. Soc. 1998; 120:12–17.

[64] Hud N.V., Downing K.H. Cryoelectron microscopy of λ phage DNA condensates in vitreous ice: the fine structure of DNA toroids. Proc. Natl. Acad. Sci. U.S.A. 2001; 98:14925–14930. 11734630 10.1073/pnas.261560398 PMC64960

[65] Sartori Blanc N., Senn A., Leforestier A., Livolant F., Dubochet J. DNA in human and stallion spermatozoa forms local hexagonal packing with twist and many defects. J. Struct. Biol. 2001; 134:76–81. 11469879 10.1006/jsbi.2001.4365

[66] Yasar S., Podgornik R., Valle-Orero J., Johnson M.R., Parsegian V.A. Continuity of states between the cholesteric → line hexatic transition and the condensation transition in DNA solutions. Sci. Rep. 2014; 4:6877. 25371012 10.1038/srep06877 PMC4220286

[67] Feughelman M., Langridge R., Seeds W.E., Stokes A.R., Wilson H.R., Hooper C.W., Barclay R.K., Hamilton L.D. Molecular structure of deoxyribose nucleic acid and nucleoprotein. Nature. 1955; 175:834–838. 14370232

[68] Leforestier A, Livolant F The bacteriophage genome undergoes a succession of intracapsid phase transitions upon DNA ejection. J. Mol. Biol. 2010; 396:364–395. 10.1016/j.jmb.2009.11.047 19944702

[69] Liao L.W., Cole R.D. Condensation of dinucleosomes by individual subfractions of H1 histone. J. Biol. Chem. 1981; 256:10124–10128. 7275970

[70] Todoli S., Perez P.J., Clauvelin S., Olson W.K. Contributions of sequence to the higher-order structures of DNA. Biophys. J. 2018; 112:416–426. 10.1016/j.bpj.2016.11.017 PMC5300782 27955889

[71] Maeshima K., Ide S., Babokhov M. Dynamic chromatin organisation without the 30-nm fiber. Curr. Opin. Cell Biol. 2019; 58:95–104. 30908980 10.1016/j.ceb.2019.02.003

[72] Hergeth S.P., Schneider R. The H1 linker histones: multifunctional proteins beyond the nucleosome core particle. EMBO Rep. 2015; 16:1439–1453. 26474902 10.15252/embr.201540749 PMC4641498

[73] Eltsov M., Maclellan A.M., Maeshima K., Frangakis A.S., Dubochet J. Analysis of cryo-electron microscopy images does not support the existence of 30-nm fibres in mitotic chromosomes in situ. Proc. Natl. Acad. Sci. U.S.A. 2008; 105:19732–19737. 19064912 10.1073/pnas.0810057105 PMC2604964

[74] Daban J.R. Electron microscopy and atomic force microscopy studies of chromatin and metaphase chromatin structure. Micron. 2011; 42:733–750. 21703860 10.1016/j.micron.2011.05.002

[75] Hill C.S., Rimmer J.M., Green B.N., Finch J.T., Thomas J.O. Histone-DNA interactions and their modulation by phosphorylation of –Ser-Pro-X-Lys/Arg- motifs. EMBO J. 1991; 10:1939–1948. 2050127 10.1002/j.1460-2075.1991.tb07720.x PMC452869

[76] Sung M.T., Freedlender E.F. Sites of in vivo phosphorylation of histone H5. Biochemistry. 1978; 17:1884–1890. 656368 10.1021/bi00603a013

[77] Panchenko T., Sorensen T.C., Woodcock C.L., Kan Z.Y., Wood S., Resch M.G., Luger K., Englander S.W., Hansen J.C., Black B.E. Replacement of histone H3 with CENP-A directs global nucleosome array condensation and loosening of nucleosome superhelical termini. Proc. Natl. Acad. Sci. U.S.A. 2011; 108:16588–16593. 21949362 10.1073/pnas.1113621108 PMC3189058

[78] Roulland Y., Ouararhni K., Naidenov M., Ramos L., Shuaib M., Syed S.H., Lone I.N., Boopathi R., Fontaine E., Papai G. et al. The flexible ends of the CENP-A nucleosome are required for mitotic fidelity. Mol. Cell. 2016; 63:674–685. 27499292 10.1016/j.molcel.2016.06.023

[79] Doyen C.M., Montel F., Gautier T., Menoni H., Claudet C., Delacour-Larose M., Angelov D., Hamiche A., Bednar J., Faivre-Moskalenko C. et al. Dissection of the unusual structural and functional properties of the variant H2A.Bbd nucleosome. EMBO J. 2006; 25:4234–4244. 16957777 10.1038/sj.emboj.7601310 PMC1570437

[80] Arimura Y., Kimura H., Oda T., Sato K., Osakabe A., Tachiwana H., Sato Y., Kinugasa Y., Ikura T., Sugiyama M. et al. Structural basis of a nucleosome containing histone H2A.B/H2A.Bbd that transiently associates with reorganized chromatin. Sci. Rep. 2013; 3:3510. 24336483 10.1038/srep03510 PMC3863819

[81] Shukla M.S., Syed S.H., Goutte-Gattat D., Richard J.L., Montel F., Hamiche A., Travers A., Faivre-Moskalenko C., Bednar J., Hayes J.J. et al. The docking domain of histone H2A is required for H1 binding and RSC-mediated nucleosome remodelling. Nucleic Acids Res. 2011; 39:2559–2570. 21131284 10.1093/nar/gkq1174 PMC3074127

[82] Woodcock C.L., Grigoryev S.A., Horowitz R.A., Whitaker N. A chromatin folding model that incorporates linker variability generates fibers resembling the native structures. Proc. Natl. Acad. Sci. U.S.A. 1993; 90:9021–9025. 8415647 10.1073/pnas.90.19.9021 PMC47493

[83] Daban J.R., Bermúdez A. Interdigitated solenoid model for compact chromatin fibres. Biochemistry. 1998; 37:4299–4304. 9556343 10.1021/bi973117h

[84] Kepper N, Foethke D, Stehr R., Wedemann G., Rippe K. Nucleosome geometry and internucleosomal interactions control the chromatin fiber conformation. Biophys. J. 2008; 95:3692–3705. 18212006 10.1529/biophysj.107.121079 PMC2553103

[85] Wong H., Victor J.M., Mozziconacci J. An all-atom model of the chromatin fiber containing linker histones reveals a versatile structure tuned by the nucleosomal repeat length. PLoS One. 2007; 2:e877. 17849006 10.1371/journal.pone.0000877 PMC1963316

[86] Koslover E.F., Fuller C.J., Straight A.F., Spakowitz A.J. Local geometry and elasticity in compact chromatin structure. Biophys. J. 2010; 99:3941–3950. 21156136 10.1016/j.bpj.2010.10.024 PMC3000514

[87] Eltsov M., Grewe D., Lemercier N., Frangakis A., Livolant F., Leforestier A. Nucleosome conformational variability in solution and in interphase nuclei evidenced by cryoelectron microscopy of vitreous sections. Nucleic Acids Res. 2018; 46:9189–9200. 30053160 10.1093/nar/gky670 PMC6158616

[88] Norouzi D., Katebi A., Cui F., Zhurkin V.B. Topological diversity of chromatin fibrers: interplay between nucleosome repeat length, DNA linking number and the level of transcription. AIMS Biophys. 2015; 2:613–629. 28133628 10.3934/biophy.2015.4.613 PMC5271602

[89] Bartolomé S., Bermúdez A., Daban J.R. Internal structure of the 30 nm chromatin fiber. J. Cell Sci. 1994; 107:2983–2992. 7698998 10.1242/jcs.107.11.2983

[90] Müller U., Zentgraf H., Eicken I., Keller W. Higher order structure of simian virus 40 chromatin. Science. 1978; 201:406–415. 208155 10.1126/science.208155

[91] Germond J.E., Hirt B., Oudet P., Gross-Bellark M., Chambon P. Folding of the DNA double helix in chromatin-like structures from simian virus 40. Proc. Natl. Acad. Sci. U.S.A. 1975; 72:1843–1847. 168578 10.1073/pnas.72.5.1843 PMC432643

[92] Naughton C., Avlonitis N., Corless S., Prendergast J.G., Mati I.K., Eijk P.P., Cockroft S.L., Bradley M., Ylstra B., Gilbert N. Transcription forms and remodels supercoiling domains unfolding large-scale chromatin structures. Nat. Struct. Mol. Biol. 2013; 20:387–395. 23416946 10.1038/nsmb.2509 PMC3689368

