# Supplementary material for: Modelling and DNA topology of compact 2-start and 1-start chromatin fibres
Source: Nucleic Acids Res. 2019 Jun 20;47(18):9902–24. doi: 10.1093/nar/gkz495 (PMC6765122; doi:10.1093/nar/gkz495)
Supplement: gkz495_Supplemental_Files [file gkz495_supplemental_files.zip › Supplementary material.pdf]

SUPPLEMENTARY MATERIAL

S1. Writhing of fibre DNA in response to linker torsion.

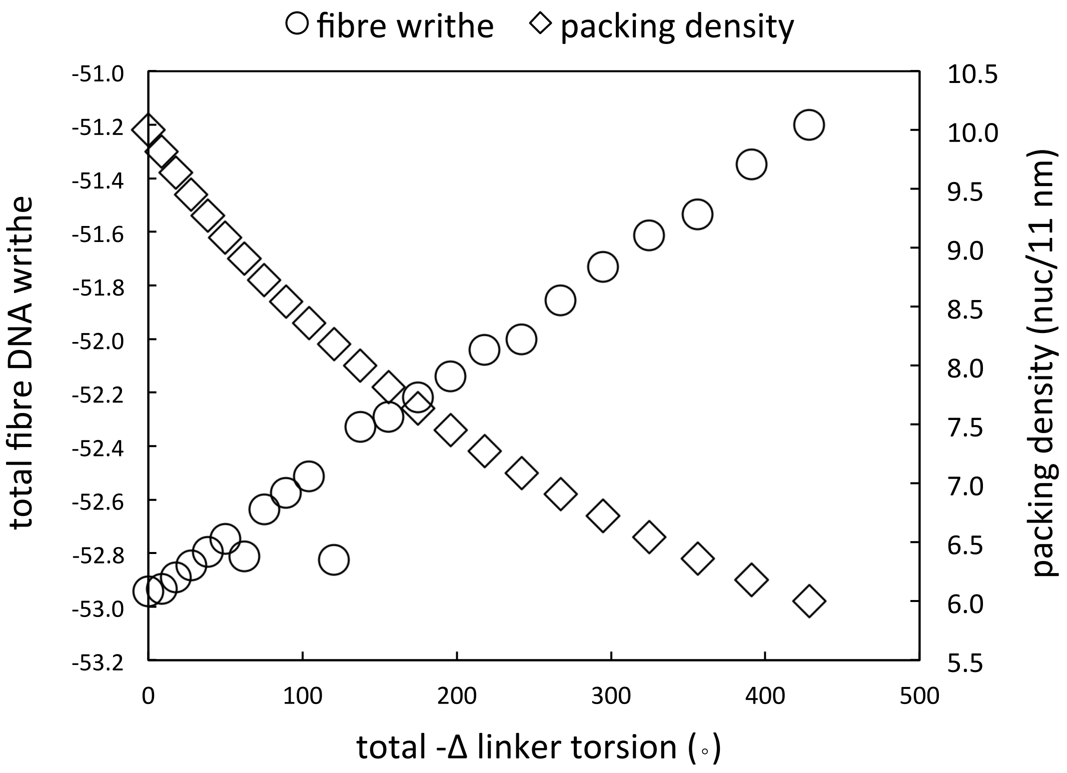

The 1-start crossed linker fibre with the 197 bp NRL was constructed in a closed system (see movie S3 in SUPPLEMENTARY DATA). The writhe of fibre DNA at varying compaction states is plotted against the torsion generated by the linker DNA using the relative rotation between consecutive nucleosomes as a proxy. The plot demonstrates that increased fibre coiling containing negative writhe is accompanied by the overtwisting (positive) of linkers.

S2. Estimation of the intrinsic helical repeat in linker DNA

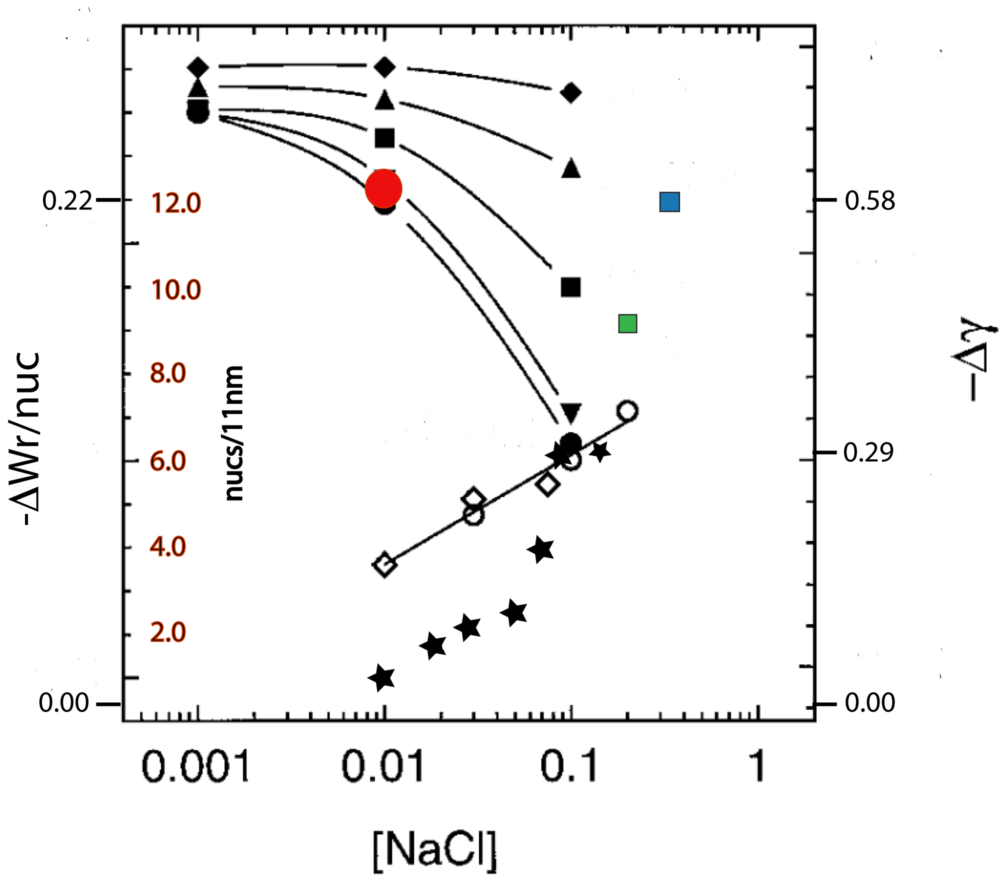

Change in linker DNA helical repeat with fibre compaction. Symbols as in Figure 12.

To estimate the intrinsic helical repeat of linker DNA in 1-start fibres we assumed that the full compaction from an extended array was a direct twist to writhe compensation and was influenced by both the electrolyte cation and the C-terminal tail of the linker histone. The difference in the writhe between a 1-start fibre and an extended array with an NRL of 207 bp (i.e. a linker DNA of 40 bp) and containing linker histone H5 is –(1.68-1.46)/nuc = -0.22/nuc, where the value of -1.46 is that calculated for a chromatosome (Figure 6) and -1.68 for the corresponding 1-start fibre when $\Delta Tw=0$. The value of -1.46 is similar to that of ~-1.40 computed by Le Bret (1988) as the most probable value for a nucleosome with a fixed left-handed crossover bound to a minicircle. The scale for $-\Delta\gamma$ was then adjusted accordingly from Figure 12 as shown above. The zero values represent the baselines for changes in writhe and helical repeat. On this basis the reduction in the helical repeat of linker DNA on compaction is 0.58 bp/turn. This value is consistent with an approximate value of 2 bp/linker calculated by Barbi et al (2014) for a fibre with a similar length linker.

The exact value of the helical repeat of DNA in solution depends on the ionic conditions has been variously estimated experimentally to be 10.4 bp/turn (Wang, 1979; Peck and Wang, 1980), 10.5-10.6 bp/turn (Richardson et al., 1988), and 10.6 bp/turn (Rhodes and Klug, 1980) and theoretically to be 10.6 bp/turn (Levitt, 1978). Taking these numbers as a baseline our calculation estimates that the helical repeat of linker DNA in a compact 1-start fibre is in the range of 9.82-10.02 bp/turn. Assuming that all the linker DNA (i.e. 60 bp for an NRL of 207 bp) is overwound the range is ~9.90-10.2 bp/turn.

We note that these considerations apply to dilute aqueous conditions. In vivo molecular crowding and a possible non-zero superhelical density of DNA would mean that the baseline helical repeat for an extended array would differ.

S3. Condensation of the 30-nm fibre

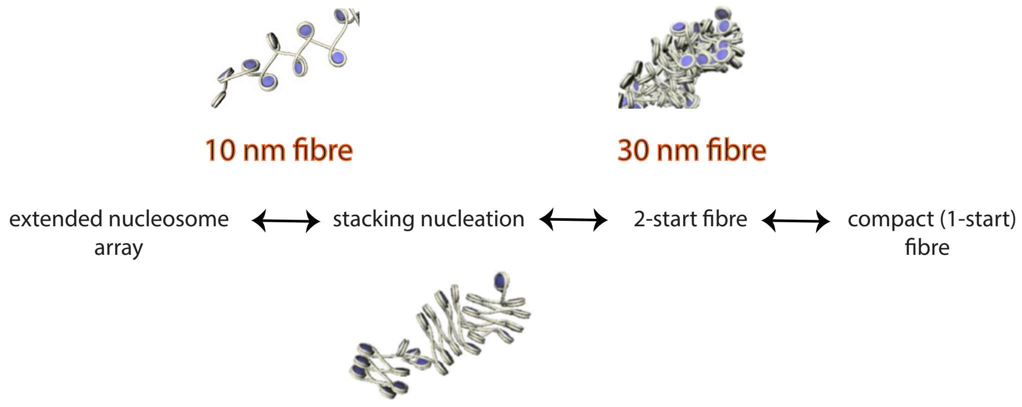

Schematic of fibre condensation process.

The schematic distinguishes three different stages in the folding of a 30-nm fibre with longer NRLs, consistent with the conclusions of Smirnov et al. (1988) who suggested that the process consisted of at least two steps. The dependence of the initial compaction of chicken erythrocyte fibres (Gerchmann and Ramakrishnan, 1987) on sodium ion concentration has approximately the same slope as the change in the DNA helical repeat over the same concentration range. This implies that the electrolyte cation concentration dominates in the initial coiling of the 10-nm fibre. We suggest that the inflection point corresponds to the nucleation of stacking observed by Scheffer et al (2012) and that the subsequent change in slope reflects both the functional engagement of the linker histone C-terminal tail and the establishment of topological microdomains (Travers and Muskhelishvili,1998) by internucleosomal contacts. A change in folding characteristics of the fibre at a similar Na^{+} concentration of 40-50 mM has been previously noted (Thomas and Butler, 1980).

Interdigitation of a 2-start to a 1-start structure requires a sufficient distance between adjacent nucleosomes in the 2-start stacks – for example, as in Scheffer et al (2011).

References

Barbi,M., Mozziconacci,J., Wong,H. and Victor,J.M. (2014) DNA topology in chromosomes: a quantitative survey and it physiological implications. J. Math. Biol. 68, 145-179.

Gerchman,S.E. and Ramakrishnan,V. (1987) Chromatin higher-order structure studied by neutron scattering and scanning transmission electron microscopy. Proc. Natl. Acad. Sci. U.S.A., 84, 7802-7806.

Le Bret,M. 1988. Computation of the helical twist of nucleosomal DNA. J. Mol. Biol. 200, 285–290.

Levitt, M. (1978) How many base-pairs per turn does DNA have in solution and in chromatin? Some theoretical calculations. Proc. Natl. Acad. Sci. U.S.A. 75, 640-644.

Peck,L.J and Wang,J.C. (1980) Sequence dependence of the helical repeat of DNA in solution. Nature. 292, 375-378.

Rhodes,D. and Klug,A. (1980) Sequence-dependent helical periodicity of DNA. Nature. 292, 378-380.

Richardson,S.M., Boles,T.C and Cozzarelli,N.R. (1988) The helical repeat of underwound DNA in solution. Nucleic Acids Res. 16, 6607-6616.

Scheffer,M.P., Eltsov,M. and Frangakis,A.S. (2011) Evidence for short-range helical order in the 30-nm chromatin fibres of erythrocyte nuclei. Proc. Natl. Acad. Sci. U.S.A., 108, 16992–16997.

Scheffer,M.P., Eltsov,M.,Bednar,J. and Frangakis,A.S. (2012) Nucleosomes stacked with aligned dyad axes are found in native compact chromatin in vitro. J. Struct. Biol. 178, 207-214.

Smirnov,I.V., Dimitrov,S.I. and Makarov,V.L. (1988) NaCl-induced chromatin condensation. Application of static light scattering at 90° and stopped flow technique. J. Biomol. Struct. Dyn. 5, 1127-1134.

Thomas,J.O. and Butler,P.J. (1980) Size-dependence of a stable higher-order structure of chromatin. J. Mol. Biol. 144, 89-93.

Travers,A. and Muskhelishvili,G (1998) DNA microloops and microdomains: a general mechanism for transcriptional activation by torsional transmission. J. Mol. Biol. 279, 1027-1043.

Wang,J.C. Helical repeat of DNA in solution. (1979) Proc. Natl. Acad. Sci. U.S.A. 76, 200-203.

Supplementary files:

Movie S1. S1.mp4 Compaction and decompaction of 2-start (left) and 1-start (right) fibres

Movie S2. S2.mp4 See text

Movie S3. S3.mp4 See text
